# A minimal model of cognition based on oscillatory and current-based reinforcement processes

**DOI:** 10.1098/rsif.2024.0402

**Published:** 2025-01-22

**Authors:** Linnéa Gyllingberg, Yu Tian, David J. T. Sumpter

**Affiliations:** ^1^ Department of Mathematics, University of California, Los Angeles, CA, USA; ^2^ Department of Mathematics, Uppsala University, Uppsala, Sweden; ^3^ Nordita, Stockholm University and KTH Royal Institute of Technology, Stockholm, Sweden; ^4^ Department of Information Technology, Uppsala University, Uppsala, Sweden

**Keywords:** minimal cognition, shortest paths, oscillations, slime mould, *Physarum polycephalum*, network

## Abstract

Building mathematical models of brains is difficult because of the sheer complexity of the problem. One potential starting point is basal cognition, which gives an abstract representation of a range of organisms without central nervous systems, including fungi, slime moulds and bacteria. We propose one such model, demonstrating how a combination of oscillatory and current-based reinforcement processes can be used to couple resources in an efficient manner, mimicking the way these organisms function. A key ingredient in our model, not found in previous basal cognition models, is that we explicitly model oscillations in the number of particles (i.e. the nutrients, chemical signals or similar, which make up the biological system) and the flow of these particles within the modelled organisms. Using this approach, our model builds efficient solutions, provided the environmental oscillations are sufficiently out of phase. We further demonstrate that amplitude differences can promote efficient solutions and that the system is robust to frequency differences. In the context of these findings, we discuss connections between our model and basal cognition in biological systems and slime moulds, in particular, how oscillations might contribute to self-organized problem-solving by these organisms.

## 1. Introduction

Understanding cognitive abilities, such as perception, problem solving and learning, is a central and long-standing question in neurobiology. One crucial aspect of cognition is the role of *oscillatory processes* [1]. In the human brain, for example, theta oscillations are linked to active learning in infants [2] and play an important role in memory formation [3]; beta oscillations have shown pivotally for multi-sensory learning [4]; alpha oscillations are central in temporal attention [5]; and cross-frequency coupling, i.e. coupling between neurons of different frequencies, has been proposed as a mechanism for working memory [6,7]. Another crucial aspect of cognitive abilities is synaptic plasticity, i.e. changing the strength of the connections between neurons based on previous activity [8,9]. Such adaptive processes, often referred to as *reinforcement*, have been studied in both experimental and computational settings. For example, a study on rats shows that reinforcement determines the timing dependence of corticostriatal synaptic plasticity *in vivo* [10], and dopamine-dependent synaptic plasticity has been studied in mice [11].

While the human brain serves as the primary model system for cognitive research, the field of basal cognition (also referred to as minimal cognition) aims to articulate the fundamental requirements necessary for the generation of cognitive phenomena [12,13]. Cognitive-like abilities are not limited to organisms with central nervous systems, but are also found in aneural organisms [14]. All living organisms employ sensory and information-processing mechanisms to assess and engage with both their internal environment and the world around them, and oscillations often play a crucial role in their function [14]. For example, self-sustained oscillations that encode various types of information have been observed in cells [15,16]. Oscillations are also found in the root apex of many plants [17] and have been suggested to be a driving mechanism for deciding where the plant should branch its roots [18].

*Physarum polycephalum* is a popular model organism for studying problem-solving in aneural organisms and better understanding basal cognition [19–21]. In its vegetative state, known as the plasmodium, this organism takes the form of a colossal, mobile cell. The plasmodium’s fundamental structure encompasses a syncytium of nuclei and an intricate intracellular cytoskeleton, forming a complex network of cytoplasmic veins. It can expand to cover extensive areas, reaching dimensions of hundreds of square centimetres, and can divide into smaller, viable subunits. When these subunits come into contact, they have the remarkable ability to fuse and share information, leading to the reformation of a giant plasmodium. These slime moulds demonstrate habituation [22] as well as anticipation [23]. Early experiments on these single cellular organisms show that they can find the shortest paths between food sources through a labyrinth [24,25]. Further experiments have shown that it can construct efficient and robust transport networks with the same topology as the Tokyo rail network [26], avoid already exploited patches [27] and solve a Towers of Hanoi maze [28].

Experiments show that partial bodies in the plasmodium of the slime mould can also be viewed as nonlinear oscillators, where the interactions between the oscillators are strongly affected by the geometry of the tube network [29,30]. Thus, both oscillatory and reinforcement processes are involved in *Physarum polycephalum*’s cognitive abilities [14]. Oscillations in slime mould take place in different parts of the organism and on multiple time scales; from short-period oscillations in the contraction cycle, to long-period oscillations in the cell cycle [31].

In this article, we look at how oscillators, coupled through current-based reinforcement, can exhibit both problem-solving (i.e. shortest path finding) abilities and long-range synchronization observed in many cognitive phenomena. Following Boussard *et al*., who emphasize the slime mould as an ideal model system for relating basal cognitive functions to biological mechanisms [14], it is such a coupling of feedback with oscillations which we refer to as minimal cognition. This definition is also in line with discussions around properties of minimal cognition for other biological systems [32–34]. We can also see our work as contributing a model of *basal cognition*, a term first introduced in a special double issue of *Philosophical Transactions of the Royal Society B* in March 2021 [13]. This emerging field, as elucidated by Lyon *et al*., underscores the presence of cognitive abilities pre-dating the evolution of nervous systems. Indeed, fig. 1 in [13] illustrates various organisms that have been studied to comprehend basal cognition, including *Bacillus subtilis* [35], the aneural placozoan *Trichoplax adhaerens* [36] and the planarian flatworm *Platyhelminthes* [37].

**Figure 1.**
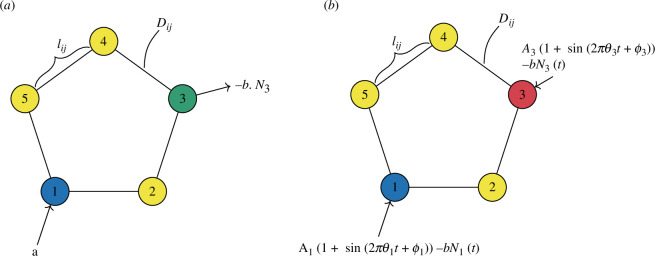
The model on a $C_{5}$ graph, where $l_{ij}$ is the length of the edge between node i and j, $D_{ij}$ is the conductivity (thickness) of the edge between i and j. In (*a*), the nodes are non-oscillating, and node 1 is a source, with an inflow at rate a and node 3 is a sink with an outflow at rate $bN_{3}$. In (*b*), the nodes are oscillating as $A_{1}(1+\sin(2\pi \theta_{1}t+\phi_{1}))-bN_{1}(t)$ and $A_{3}(1+\sin(2\pi \theta_{3}t+\phi_{3}))-bN_{3}(t)$ at node 1 and 3 respectively, meaning that the different colours of the oscillating nodes indicates different amplitudes, frequencies and phases in the oscillators.

Aligning with the broader scope of basal cognition, we aim here to define a mathematical model that elucidates the role of oscillations and reinforcement mechanisms for cognitive phenomena in aneural settings. We apply Beer’s four steps for theory creation around cognitive phenomena [38] to basal cognition. In this introduction, as a first step, we have presented a conceptual framework, i.e. the idea that reinforcement and oscillations are sufficient to produce some basal cognitive abilities. Beer’s second step, which we perform in §2, is to present a mathematical model based on oscillators and reinforcement on networks. As a third step, in §3, we study the mathematical properties of this model with a particular focus on what types of oscillations produce basal cognitive phenomena in aneural organisms. Finally, the fourth step discusses how our model might be further developed in light of the results and the degree to which they help us to understand biological systems.

From a mathematical point of view, in this article, we start by studying the dynamics of a small network, a cycle graph with two oscillators. We show that this model can generate shortest path networks, localized oscillatory behaviour and global oscillatory dynamics. Then, addressing questions raised by Boussard *et al*. [14], we look at how phase, frequency and amplitude differences can be used to induce dynamic problem-solving. Finally, we look at how larger networks of coupled oscillators can create complex networks of connections that mimic some notion of basal cognition.

## 2. Model

Our aim is to define a model which has a combination of reinforcement and oscillatory processes, which can both ‘solve’ the problem of finding the shortest path between two or more points and exhibit long-range oscillations. We abstract away from the specific biological systems and find a model of basal (or minimal) cognition [13]. To this end, we describe the motion of particles which, depending on the system, might be nutrients, chemical signals (e.g. in slime moulds [14] and many fungi [39]) or information within a decentralized system (e.g. macromolecular networks in microorganisms [40]). The system itself is modelled as a network or a graph over which the particles move. The particles travel on the edges of the graph and in doing so produce current reinforcement. The coupling between the system and its environment is modelled through an inflow and outflow of particles at some of the points on the network. To capture oscillations, which are also widespread in systems of microbes [41], slime moulds [14] and fungi [39], we will make this inflow and outflow of particles change over time.

### 2.1. Previous models

We build primarily on the work of Tero *et al*. in modelling slime mould [42]. They model the movement of particles, which are nutrient and chemical signals, on nodes on a graph, which can be thought of as the cytoskeleton of the plasmodium. The dynamic weights on the edges of the graph are the thickness of the tubes within that form the cytoskeleton. Similar analogies can be made for other basal cognitive systems. For example, for fungi, the graph is the hyphae, and the particles are nutrients [39].

Tero *et al*.’s model uses an analogy to electrical networks, to describe a flux of particles, $Q_{ij}$, between the nodes. Equations for the evolution of the conductivity of the edges, $D_{ij}(t)$ (i.e. the thickness of the edges) are then proposed as follows:


(2.1) $$\frac{dD_{ij}(t)}{dt}=f(|Q_{ij}|)-\lambda D_{ij},$$

where f is a non-decreasing function. The equation above states that edges in the tubular network are thickened if there is a sufficient volume flow and diminished otherwise. In the case where f(q)=q, this model has been proven to eventually converge to the shortest path between food sources, independently of the network structure [43,44]. In this work, the flow of particles is assumed to be in a steady state, i.e. $Q_{ij}=N_{i}-N_{j}=0$, where $N_{i}$ is the number of particles. In their work, Tero *et al*. assume that all flow between all nodes, except for the input and output nodes, is zero. They also assume that the total flux of the system is constant, meaning that the sum of the input and output is always zero. Another adaptation of the Tero *et al*. model has been presented by Ma *et al*., who introduced a stochastic version of the current reinforcement model, where the flow of the particles between the nodes is not assumed to be in the steady state [45], and (in simulations) the model also finds the shortest paths between food sources.

Mathematical models of coupled oscillators, such as the Kuramoto model [46], have played a pivotal role in investigating the dynamics of neuronal processes within the brain [47,48]. And, while it has been adapted to include synaptic plasticity, by evolving coupling strength based on phase differences and Hebbian learning principles [49–51], these models do not incorporate the current reinforcement mechanism, which is central to problem-solving by slime moulds outlined in the previous paragraph. Indeed, while the Kuramoto model has been used to model the anticipation of periodic events in slime moulds [23], there is a lack of models using coupled oscillators to study other problem-solving abilities of slime moulds, such as finding shortest paths. Alim *et al*. have made an interesting starting point into this, by studying the mechanism of signalling propagation in slime moulds [52]. Using the Stokes equations to model the cytoplasmic flow velocity with the active tension of the walls of the slime mould oscillating, they argue that their model will obey similar dynamics to the Tero *et al*. model [42], and thus lead to shortest paths solutions.

Watanabe and Takamatsu have also studied an algorithm based on the current reinforcement model, but with oscillatory inputs and outputs [53]. In this model, the nodes switch between being inputs and outputs explicitly. In terms of other applications, Ben-Ami *et al*. have recently studied a model of the flow of blood through a series of cylindrical capillaries that form a three-node network, which gives rise to self-sustained oscillations [54]. What is missing from these models, however, is an explicit representation of the number of particles on a node in combination with oscillations. It is such a model we now define.

### 2.2. Model definition

Our model consists of an undirected network, G(V,E), where V={1,2,…,n} is the node-set, and an edge (i,j)∈E is an unordered pair of two distinct nodes in the set V, where each edge (i,j) is associated with a length $l_{ij}$ ($l_{ij}=\infty$ if there is no edge). We denote the number of particles at each node i by $N_{i}$. For each edge (i,j), there is a current of particles inversely proportional to the length of the connection, $\left|N_{i}-N_{j}\right|/l_{ij}$. The edge is also characterized by its conductivity (corresponding to the thickness of the tubes in slime mould), $D_{ij}$, which will also affect the current. The number of particles at a node i will change based on the flow of the particles from the neighbouring nodes of i, j∈Γ(i), where Γ(i) represents the set of neighbouring nodes of i, according to the following equation:


(2.2) $$\frac{dN_{i}(t)}{dt}=\sum_{j\in \Gamma (i)}\frac{N_{j}(t)-N_{i}(t)}{l_{ij}}D_{ij}(t),$$

(ignoring input and output nodes for now). The conductivity of each edge (i,j)∈E changes based on the following equation:


(2.3) $$\begin{array}{r} \frac{dD_{ij}(t)}{dt}=q\frac{\left|N_{i}(t)-N_{j}(t)\right|}{l_{ij}}D_{ij}(t)-\lambda D_{ij}(t). \end{array}$$

Here, q is the reinforcement strength and λ is the decay rate. Note that, unlike previous deterministic models of current reinforcement [42,55,56], we give dynamic equations for the number of particles at each node, as we do not assume the flow of particles to be in equilibrium on each edge as in [42]. This approach is similar to that taken by [45], who looked at a stochastic model of current reinforcement similar to the one we study here. Another difference to much of the work by Tero and co-workers is that we only consider the case where the increase in conductivity is proportional to (and not a nonlinear function of) the current. Although we do not explicitly write down the equations for the particle flow on the edges, the rate at which particles are transported from one node to another can be derived in the same way as done by Tero *et al*. [42].

In the above description, there is no inflow or outflow of particles in the system and thus no interaction between the system and the environment. We incorporate such interactions in two different cases: (i) the non-oscillatory case, where particles continuously enter the network at sources and leave at sinks with fixed rates and (ii) the oscillatory case, where the sources and sinks change over time, in the sense that sources can become sinks and vice versa. We now describe each of these in more detail.

### 2.3. Non-oscillatory sources and sinks

We start by studying non-oscillatory sources and sinks. In this setting, particles enter the network at some source(s) $S=\{s_{1},\ldots \}\subset V$ with a constant rate a, and leave the network at some sink(s) $T=\{t_{1},\ldots \}\subset V$ with a rate $bN_{i}$ for each sink i, where b is a constant. Thus, the equations describing the number of particles at each node i are given by


(2.4) $$\begin{array}{r} \frac{dN_{i}(t)}{dt}=\left\{ \begin{array}{ll} a+\sum_{j\in \Gamma (i)}\frac{N_{j}(t)-N_{i}(t)}{l_{ij}}D_{ij}(t),\; & \text{if }i\in S,\\ -bN_{i}(t)+\sum_{j\in \Gamma (i)}\frac{N_{j}(t)-N_{i}(t)}{l_{ij}}D_{ij}(t),\; & \text{if }i\in T,\\ \sum_{j\in \Gamma (i)}\frac{N_{j}(t)-N_{i}(t)}{l_{ij}}D_{ij}(t),\; & \text{otherwise}. \end{array}\right. \end{array}$$

We start by studying these equations in the case of a cyclic graph of five nodes, $C_{5}$. Here, the nodes are labelled (1,2,3,4,5), and node 1 is a source, with an inflow a, while node 3 is a sink, with outflow $-bN_{3}$ at that node (figure 1*a*).

### 2.4. Oscillatory nodes

To incorporate oscillations, we consider nodes which alternate between being sources and sinks. For these nodes, the rate at which particles enter or leave the network through the environment changes over time. To this end, we model the interaction with the environment as the following function, for each node i∈O:


(2.5) $$\begin{array}{lrr} & A_{i}(1+\sin(2\pi \theta_{i}t+\phi_{i}))-bN_{i}(t), & \end{array}$$

where O denotes the set of oscillatory nodes, and $A_{i},\theta_{i},\phi_{i}$ characterize the features of the environment around node i. Specifically, $A_{i}$ is the amplitude, $\theta_{i}$ is the frequency, $\phi_{i}$ is the phase, and b is the output rate. We choose parameters for the function (equation (2.5)), so it can have both positive and negative values at varying time t, to model that particles can both enter and leave the network at node i. For $C_{5}$, we let nodes 1 and 3 be the oscillatory nodes, i.e. O={1,3} (figure 1*b*).

We choose this function to ensure that the model reflects the dynamic behaviour of slime moulds, which do not conserve mass but constantly grow and reorganize. By designing $A_{i}(1+\sin(2\pi \theta_{i}t+\phi_{i}))$ to be non-negative, we ensure a positive inflow, while $-bN_{i}(t)$ manages the outflow, preventing any negative number of particles at any node at any time step. This self-stabilizing mechanism couples a positive inflow with a negative outflow, ensuring the system remains balanced. Unlike the model by Watanabe and Takamatsu, where switching is manually controlled, our model allows for spontaneous, self-organizing and autonomous switching, reflecting more natural, spontaneous transitions.

When the phase difference between two nodes is π, the sine waves corresponding to the two nodes have exactly opposite signs. In this case, when one node has particles entering the network, the other node has particles leaving it, and vice versa. To model this, we set $\phi_{1}=0$ and $\phi_{3}=\pi$, while we set the other parameters to be identical for both nodes, i.e. $A_{1}=A_{3}=A$ and $\theta_{1}=\theta_{3}=\theta$. Hence, the evolution of $N_{1}$ and $N_{3}$ is


(2.6) $$\begin{array}{r} \left\{ \begin{array}{ll} \frac{dN_{1}(t)}{dt} & =A(1+\sin(2\pi \theta t))-bN_{1}(t)+\frac{\left(N_{5}(t)-N_{1}(t)\right)}{l_{51}}D_{51}+\frac{\left(N_{2}(t)-N_{1}(t)\right)}{l_{12}}D_{12}\\ \;\frac{dN_{3}(t)}{dt} & =A(1+\sin(2\pi \theta t+\pi ))-bN_{3}(t)+\frac{\left(N_{2}(t)-N_{3}(t)\right)}{l_{23}}D_{23}+\frac{\left(N_{4}(t)-N_{3}(t)\right)}{l_{34}}D_{34} \end{array}\right., \end{array}$$

while others maintain the same as in equation (2.4).

Building on this special case, we now allow the phase of node 3 to take values between −π and π, i.e. $\phi_{3}=\phi \in \left(-\pi ,\pi \right]$. Similarly, the amplitude, which corresponds to the number of particles waiting at one node to enter the network and also the capacity of the node for them to enter the network, is set to $A_{1}=A$ and $A_{3}=\alpha A$, where $\alpha =A_{3}/A_{1}$ encodes the amplitude ratio. Similarly, the frequency, which corresponds to how fast the environment changes around each node, is set to $\theta_{1}=\theta$ and $\theta_{3}=\gamma \theta$, where $\gamma =\theta_{3}/\theta_{1}$ encodes the frequency ratio. The evolution of $N_{1}$ and $N_{3}$ is then modified to be


(2.7) $$\begin{array}{r} \left\{ \begin{array}{ll} \frac{dN_{1}(t)}{dt} & =A(1+\sin(2\pi \theta t))-bN_{1}(t)+\frac{\left(N_{5}(t)-N_{1}(t)\right)}{l_{51}}D_{51}+\frac{\left(N_{2}(t)-N_{1}(t)\right)}{l_{12}}D_{12}\\ \frac{dN_{3}(t)}{dt} & =\alpha A(1+\sin(2\pi \gamma \theta t+\phi ))-bN_{3}(t)+\frac{\left(N_{2}(t)-N_{3}(t)\right)}{l_{23}}D_{23}+\frac{\left(N_{4}(t)-N_{3}(t)\right)}{l_{34}}D_{34} \end{array}\right., \end{array}$$

while the other equations maintain the same as in equation (2.4).

### 2.5. Large graphs

**Figure 2.**
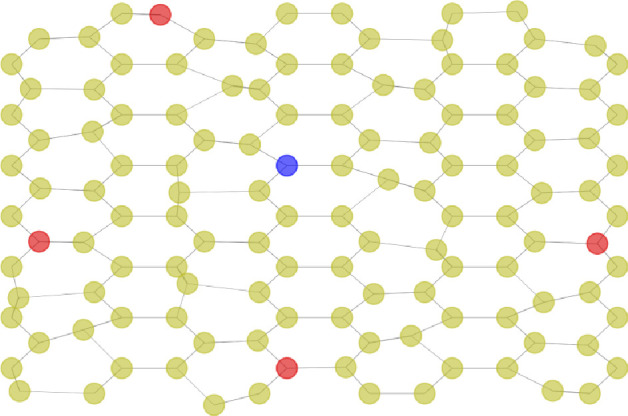
Example of a large regular graph of degree 3, where the node in blue corresponds to an oscillatory node with phase 0 and the nodes in red correspond to oscillatory nodes with phase π in later simulations.

The simplest graph for two nodes to have at least two paths connecting them is a cycle graph, and the smallest one to have two paths of different lengths while the two nodes are not directly connected is a cycle graph of length 5; thus $C_{5}$ is selected to illustrate our model in the previous sections. There are many ways to generalize this to larger graphs, where e.g. we can consider cycle graphs of arbitrary sizes (larger than 5), but there are still only two paths connecting the nodes. To systematically incorporate more complexity into the graph, we consider regular graphs of degree 3, meaning that each node has three neighbours instead of two. This choice is inspired by the observation that, as with many other biological networks, the average degree of a node in *Physarum polycephalum* networks is approximately three, with only minor variations due to end branches [57,58]. For the graph to have a clear geometric meaning, we choose nodes and edges to be the hexagonal tiling of the plane, leading to the hexagonal lattice graph. We have also included a small level of noise to the position of some nodes so that paths between the nodes are mostly of different lengths (see figure 2 for example). In this way, we have more geometrically meaningful paths connecting the nodes, which better models biological systems.

## 3. Results

We now show that our proposed model can find the shortest path in the cycle graph of size 5 for a fixed source and sink, in the sense that at steady state conductivity is non-zero only on edges on the shortest path between the source and sink. We then show, in §3.2, that this feature is maintained in simulations in the oscillatory setting when the phase difference is maximized, and further investigate the role of phase, amplitude and frequency. Finally, in §3.3, we look at larger graphs with multiple oscillating nodes. Throughout this section, we maintain our assumption that in the change of conductivity, the reinforcement effect, as parametrized in q, should be orders of magnitude larger than the decaying effect, as parametrized in λ, over time. Specifically, in the numerical simulations, we choose q/λ=10. Similar results will be obtained when we change q but maintain a similar ratio and similar final results will be reached when we choose the ratio to be much larger, but after a longer time.

### 3.1. Non-oscillatory sources and sinks

Our model on $C_{5}$ (shown in figure 1*a*) is described by the following set of equations:


(3.1) $$\begin{array}{r} \begin{array}{rl} \frac{dN_{1}(t)}{dt} & =a+\frac{\left(N_{2}-N_{1}\right)}{l_{12}}D_{12}+\frac{\left(N_{5}-N_{1}\right)}{l_{51}}D_{51},\\ \frac{dN_{2}(t)}{dt} & =\frac{\left(N_{1}-N_{2}\right)}{l_{12}}D_{12}+\frac{\left(N_{3}-N_{2}\right)}{l_{23}}D_{23},\\ \frac{dN_{3}(t)}{dt} & =-bN_{3}+\frac{\left(N_{2}-N_{3}\right)}{l_{23}}D_{23}+\frac{\left(N_{4}-N_{3}\right)}{l_{34}}D_{34},\\ \frac{dN_{4}(t)}{dt} & =\frac{\left(N_{3}-N_{4}\right)}{l_{34}}D_{34}+\frac{\left(N_{5}-N_{4}\right)}{l_{45}}D_{45},\\ \frac{dN_{5}(t)}{dt} & =\frac{\left(N_{1}-N_{5}\right)}{l_{51}}D_{51}+\frac{\left(N_{4}-N_{5}\right)}{l_{45}}D_{45},\\ \frac{dD_{12}(t)}{dt} & =q\frac{\left|N_{1}-N_{2}\right|}{l_{12}}D_{12}-\lambda D_{12},\\ \frac{dD_{23}(t)}{dt} & =q\frac{\left|N_{2}-N_{3}\right|}{l_{23}}D_{23}-\lambda D_{23},\\ \frac{dD_{34}(t)}{dt} & =q\frac{\left|N_{3}-N_{4}\right|}{l_{34}}D_{34}-\lambda D_{34},\\ \frac{dD_{45}(t)}{dt} & =q\frac{\left|N_{4}-N_{5}\right|}{l_{45}}D_{45}-\lambda D_{45},\\ \frac{dD_{51}(t)}{dt} & =q\frac{\left|N_{5}-N_{1}\right|}{l_{51}}D_{51}-\lambda D_{51}. \end{array} \end{array}$$

To derive the equilibrium points, we set all time derivatives in the system equation (3.1) to zero. We solve the resulting system of algebraic equations by sequentially isolating the variables for concentrations $N_{i}$ and conductivities $D_{ij}$, recognizing that, at equilibrium, the net flux between any two connected nodes that are neither sources nor sinks must be zero. This approach yields different possible solutions based on which pathways have non-zero conductivities. We derive the first two equilibrium points, $E_{1}$ and $E_{2}$, by considering cases where flow is confined to distinct paths: 1−2−3 or 1−5−4−3, respectively. The equilibrium points are given by


$$\begin{array}{rl} E_{1} & =(N_{1}^{*},N_{2}^{*},N_{3}^{*},N_{4}^{*},N_{5}^{*},D_{12}^{*},D_{23}^{*},D_{34}^{*},D_{45}^{*},D_{51}^{*})\\ & =\left(\frac{a}{b}+\frac{\lambda l_{23}}{q}+\frac{\lambda l_{12}}{q},\frac{a}{b}+\frac{\lambda l_{23}}{q},\frac{a}{b},N_{4}^{*},N_{5}^{*},\frac{aq}{\lambda },\frac{aq}{\lambda },0,0,0\right) \end{array}$$

and


$$\begin{array}{rl} E_{2} & =(N_{1}^{*},N_{2}^{*},N_{3}^{*},N_{4}^{*},N_{5}^{*},D_{12}^{*},D_{23}^{*},D_{34}^{*},D_{45}^{*},D_{51}^{*})\\ & =\left(\frac{a}{b}+\frac{\lambda l_{34}}{q}+\frac{\lambda l_{45}}{q}+\frac{\lambda l_{51}}{q},N_{2}^{*},\frac{a}{b},\frac{a}{b}+\frac{\lambda l_{34}}{q},\frac{a}{b}+\frac{\lambda l_{34}}{q}+\frac{\lambda l_{45}}{q},0,0,\frac{aq}{\lambda },\frac{aq}{\lambda },\frac{aq}{\lambda }\right). \end{array}$$

When these paths have equal effective lengths, an additional equilibrium, $E_{3}$, arises where both paths have balanced flow, given by


$$\begin{array}{rl} E_{3} & =(N_{1}^{*},N_{2}^{*},N_{3}^{*},N_{4}^{*},N_{5}^{*},D_{12}^{*},D_{23}^{*},D_{34}^{*},D_{45}^{*},D_{51}^{*})\\ & =\left(\frac{a}{b}+\frac{\lambda l_{23}}{q}+\frac{\lambda l_{12}}{q},\frac{a}{b}+\frac{\lambda l_{23}}{q},\frac{a}{b},\frac{a}{b}+\frac{\lambda l_{34}}{q},\frac{a}{b}+\frac{\lambda l_{34}}{q}+\frac{\lambda l_{45}}{q},\frac{aq}{2\lambda },\frac{aq}{2\lambda },\frac{aq}{2\lambda },\frac{aq}{2\lambda },\frac{aq}{2\lambda }\right). \end{array}$$

The derivation requires examining these cases to fully describe all potential steady states. For the detailed step-by-step process, we refer to appendix A. The three different combinations of steady states are shown in figure 5.

**Figure 3.**
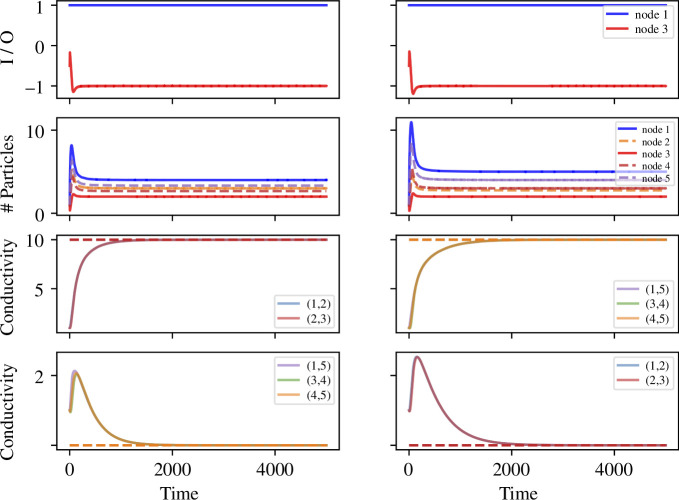
Results from the model on a $C_{5}$ graph, with node 1 as a source, node 3 as a sink, an inflow at rate a=1, an outflow at rate b=0.5 and the lengths of all edges are $l_{ij}=10$ apart from $l_{12}$. On the *left*, we set $l_{12}=10$, thus the path going through 1−2−3 is the shortest one. On the *right*, we set $l_{12}=30$, thus the path going through 1−5−4−3 is the shortest one. The first row shows the input at node 1 and output at node 3, the second row plots the number of particles on each node, $N_{i}(t)$, the third row indicates the conductivity of the edges in the shortest path, and the last row indicates the conductivity of the remaining edges in the graph, where the dashed lines in the corresponding colour show the analytical results of the steady state.

In figure 3, we see the temporal dynamics of our model with non-oscillatory sources and sinks on the $C_{5}$ graph, for two different values of the parameter $l_{12}$. In these simulations, we set the inflow rate a=1, the outflow rate b=0.5, the parameter of reinforcement q=0.1, and the decay parameter λ=0.01. In the left panel $l_{12}=10$, meaning that 1−2−3 is the shortest path between the source and the sink. Whereas in the right panel $l_{12}=30$, meaning that 1−5−4−3 is the shortest path between the source and the sink. We observe that the conductivity of the edges in the shortest path increases over time until the steady state is reached, while the conductivity of the edges that are not in the shortest path can also increase at the beginning, but will eventually converge to 0. For the number of particles, almost all nodes have an initial accumulating period before converging to the steady states.

The Jacobian matrix of the system equation (3.1), is given by the following:


(3.2) $$\begin{array}{r} J=\left(\begin{array}{cccccccccc} -\frac{D_{12}}{l_{12}}-\frac{D_{51}}{l_{51}} & \frac{D_{12}}{l_{12}} & 0 & 0 & \frac{D_{51}}{l_{51}} & \frac{N_{2}-N_{1}}{l_{12}} & 0 & 0 & 0 & \frac{N_{5}-N_{1}}{l_{51}}\\ \frac{D_{12}}{l_{12}} & -\frac{D_{12}}{l_{12}}-\frac{D_{23}}{l_{23}} & \frac{D_{23}}{l_{23}} & 0 & 0 & \frac{N_{2}-N_{1}}{l_{12}} & \frac{N_{3}-N_{2}}{l_{23}} & 0 & 0 & 0\\ 0 & \frac{D_{23}}{l_{23}} & -b-\frac{D_{23}}{l_{23}}-\frac{D_{34}}{l_{34}} & \frac{D_{34}}{l_{34}} & 0 & 0 & \frac{N_{3}-N_{2}}{l_{23}} & \frac{N_{4}-N_{3}}{l_{34}} & 0 & 0\\ 0 & 0 & \frac{D_{34}}{l_{34}} & -\frac{D_{34}}{l_{34}}-\frac{D_{45}}{l_{45}} & \frac{D_{45}}{l_{45}} & 0 & 0 & \frac{N_{3}-N_{4}}{l_{34}} & \frac{N_{5}-N_{4}}{l_{45}} & 0\\ \frac{D_{51}}{l_{51}} & 0 & 0 & \frac{D_{45}}{l_{45}} & -\frac{D_{45}}{l_{45}}-\frac{D_{51}}{l_{51}} & 0 & 0 & 0 & \frac{N_{4}-N_{5}}{l_{45}} & \frac{N_{1}-N_{5}}{l_{51}}\\ \frac{qsgn\left(N_{1}-N_{2}\right)D_{12}}{l_{12}} & \frac{qsgn\left(N_{1}-N_{2}\right)D_{12}}{l_{12}} & 0 & 0 & 0 & \frac{q|N_{1}-N_{2}|}{l_{12}}-\lambda & 0 & 0 & 0 & 0\\ 0 & \frac{qsgn\left(N_{2}-N_{3}\right)D_{23}}{l_{23}} & \frac{qsgn\left(N_{2}-N_{3}\right)D_{23}}{l_{23}} & 0 & 0 & 0 & \frac{q|N_{2}-N_{3}|}{l_{23}}-\lambda & 0 & 0 & 0\\ 0 & 0 & \frac{qsgn\left(N_{3}-N_{4}\right)D_{34}}{l_{34}} & \frac{qsgn\left(N_{3}-N_{4}\right)D_{34}}{l_{34}} & 0 & 0 & 0 & \frac{q|N_{3}-N_{4}|}{l_{34}}-\lambda & 0 & 0\\ 0 & 0 & 0 & \frac{qsgn\left(N_{4}-N_{5}\right)D_{45}}{l_{45}} & \frac{qsgn\left(N_{4}-N_{5}\right)D_{45}}{l_{45}} & 0 & 0 & 0 & \frac{q|N_{4}-N_{5}|}{l_{45}}-\lambda & 0\\ \frac{qsgn\left(N_{5}-N_{1}\right)D_{51}}{l_{51}} & 0 & 0 & 0 & \frac{qsgn\left(N_{5}-N_{1}\right)D_{51}}{l_{51}} & 0 & 0 & 0 & 0 & \frac{q|N_{5}-N_{1}|}{l_{51}}-\lambda \end{array}\right). \end{array}$$

In the appendix A, we show that for all three steady states, this Jacobian matrix will have zero determinant, meaning that at least one eigenvalue of the Jacobian matrix will be 0. Thus, the steady states will be non-hyperbolic, implying that studying the eigenvalues of the Jacobian is not enough to determine the stability of the steady states. To do this, a full analysis of the steady states using centre manifold theory would be required [59].

Nonetheless, we can use the Jacobian matrix to understand more about what happens to the system. We note that when $D_{12}^{*}=D_{23}^{*}=0$ or $D_{34}^{*}=D_{45}^{*}=D_{51}^{*}=0$, at least one row in the Jacobian matrix will contain only zero values, and thus span a slow manifold. For the Jacobian matrix evaluated at $E_{1}$, the eigenvectors corresponding to the zero eigenvalues will be (0,0,0,1,0,0,0,0,0,0) and (0,0,0,0,1,0,0,0,0,0). This observation implies that there is a line of steady states for both $N_{4}^{*}$ and $N_{5}^{*}$, which form the slow manifold. For the Jacobian matrix evaluated at $E_{2}$, the eigenvector corresponding to the zero eigenvalue will be (0,1,0,0,0,0,0,0,0,0), corresponding to a line of steady states for $N_{2}^{*}$ which is also a slow manifold. These slow manifolds correspond to the nodes with no edges, meaning that there are particles left behind at those nodes that are stuck because the conductivity converges to 0 for the longer paths.

**Figure 4.**
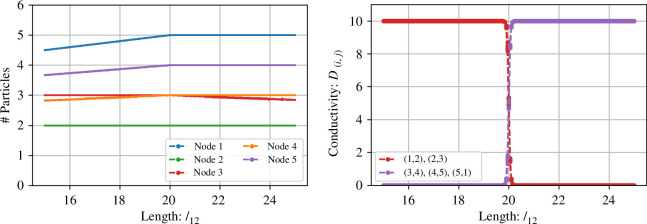
Bifurcation diagrams with $l_{12}$ as bifurcation parameter. Here $l_{23}=l_{34}=l_{45}=l_{51}=10$, a=1.0, b=0.5, q=0.1 and λ=0.01. The *left* plot shows the number of particles at each node, while the *right* plot displays the conductivity $D_{\left(i,j\right)}$ of the edges, both as a function of $l_{12}$. The diagrams are generated by simulating the equation system equation ([Disp-formula A1-E1]3.1) through 100 000 time steps, each time initiated with random initial conditions. This process is repeated 10 times for each value of $l_{12}$, with the bifurcation diagram displaying the results from the last 100 time steps of each simulation.

The numerical bifurcation diagram in figure 4 looks at the role of $l_{12}$ as a bifurcation parameter. In figure 4(left), we see that the number of particles at node 3, $N_{3}^{*}$, is always a/b, independent of the length $l_{12}$. However, $N_{1}^{*}$, $N_{4}^{*}$ and $N_{5}^{*}$ increase linearly as $l_{12}$ approaches the equilibrium point 20, after which they all are constant. $N_{2}^{*}$ is constant ($\frac{a}{b}+\frac{\lambda l_{23}}{q}=3$) until the bifurcation point $l_{12}=20$, where it instead decreases linearly. In figure 4(right), we see that the conductivity of the edges on the shortest path is always aq/λ and 0 for the edges that are not the shortest path. When the length of $l_{12}$ approaches the bifurcation point 20, the simulations have not reached a steady state after 100 000 time steps and are thus not 0 and aq/λ in the plot. When $l_{12}=20$, the two paths are of equal lengths and $D_{ij}=\frac{aq}{2\lambda }$ for all (i,j)∈E.

**Figure 5.**
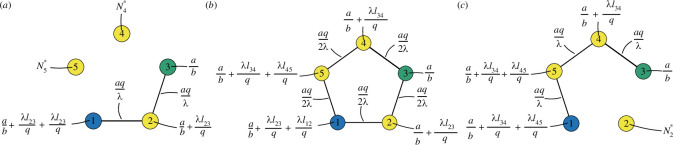
Different equilibrium points for the model on the $C_{5}$ graph with non-oscillatory sources and sinks, where node 1 (blue) is a source, and node 3 (green) is a sink. In (*a*) we see the stable equilibrium when 1−2−3 is the shortest path, in (*b*), we see the stable equilibrium when both paths have equal length, and in (*c*), we see the stable equilibrium when 1−5−4−3 is the shortest path.

Figure 5 illustrates the overall picture. In (*a*), we see that when 1−2−3 is the shortest path, there is a non-hyperbolic equilibrium where the conductivity on the edges of the longest path is zero, but the conductivity of the edges of the shortest path is $\frac{aq}{\lambda }$. The number of particles at the nodes with no connections, $N_{4}^{*}$ and $N_{5}^{*}$, spans the slow manifold. In (*b*), the paths 1−2−3 and 3−4−5−1 are of equal lengths and the conductivity of all edges is $\frac{aq}{2\lambda }$. When the path through nodes 1−2−3 is longer than 1−5−4−3 (figure 5*c*), the non-hyperbolic equilibrium point corresponds to having zero-conductivity on the edges going through node 2, and a line of steady states for the number of particles on node 2, $N_{2}^{*}$. The conductivity on the edges on the shortest path is $\frac{aq}{\lambda }$.

### 3.2. Oscillatory nodes

We now use simulations to explore the behaviour of the model with oscillatory nodes on a $C_{5}$ graph. We start from the case when the two oscillatory nodes have phase difference π (i.e. completely out of phase) and then explore the behaviour of the model when we vary the phase difference between −π and π. Finally, we look at how the amplitude and frequency of the oscillatory nodes affect the model. Throughout the section, we set nodes 1 and 3 to be the oscillatory nodes, and the length of all edges to be $l_{ij}=10$; thus, the shortest path between the two oscillatory nodes is 1−2−3.

#### 3.2.1. Phase difference

**Figure 6.**
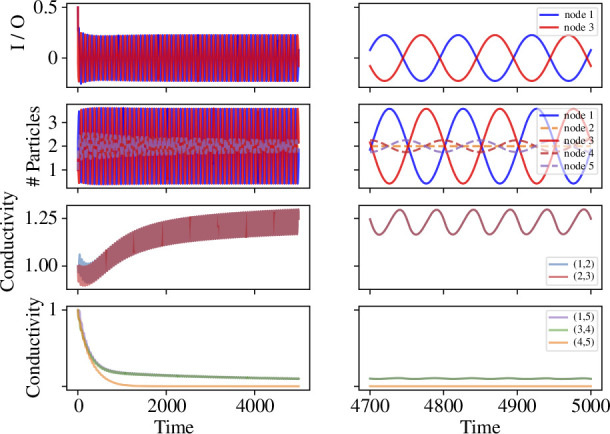
Results from the model on a $C_{5}$ graph, with nodes 1 and 3 as the oscillating nodes, rate b=0.5, amplitude $A_{1}=A_{3}=A=1$, frequency $\theta_{1}=\theta_{3}=\theta =0.01$ and phases $\phi_{1}=0$ and $\phi_{3}=\pi$, with the same reinforcement parameter, q=0.1, and decay parameter, λ=0.01, as in figure 3. The first row plots the actual input/output to the two nodes, the second row shows the number of particles in each node, the third row indicates the conductivity of the edges in the shortest path and the last row indicates the conductivity of the remaining edges in the graph, with the full profile on the *left* and the change in the long term on the *right*.

Figure 6 shows the temporal dynamics of our model when the two oscillatory nodes have phase difference π. The top two panels of figure 6 show the oscillating inputs and outputs over two different time windows. The number of particles at nodes 1 and 3 have the same frequencies as the inputs and outputs, but with a short delay as the particles flow into and out of the nodes. Oscillations with similar frequencies can be seen at nodes 4 and 5, which are on the longer path, but with much smaller amplitude and a different phase. Node 2, which is on the shortest path, reaches a steady state.

The conductivity of the edges oscillates over time, but they also change over a longer time scale. Specifically, the median conductivity of the edges on the shortest path generally increases, but with a rate of increase that tends very slowly towards 0. Interestingly, the number of particles oscillates at a frequency that is almost half that of the conductivity on the edges. This is presumably because the flow shuttles backwards and forwards. Moreover, the amplitude of the conductivity changes is much smaller than that of the oscillations of particles at nodes 1 and 3 (note the scale on the axis for conductivity in figure 6). The median conductivity of the edges that are not on the shortest path decreases over time and converges to values either 0 or very close to 0 (with no or very small oscillations) depending on the initial conditions (see the bottom row of figure 6).

We observe similar features of the model on cycles of larger sizes and also graphs of more general structure (see more details in Appendix B.2).

**Figure 7.**
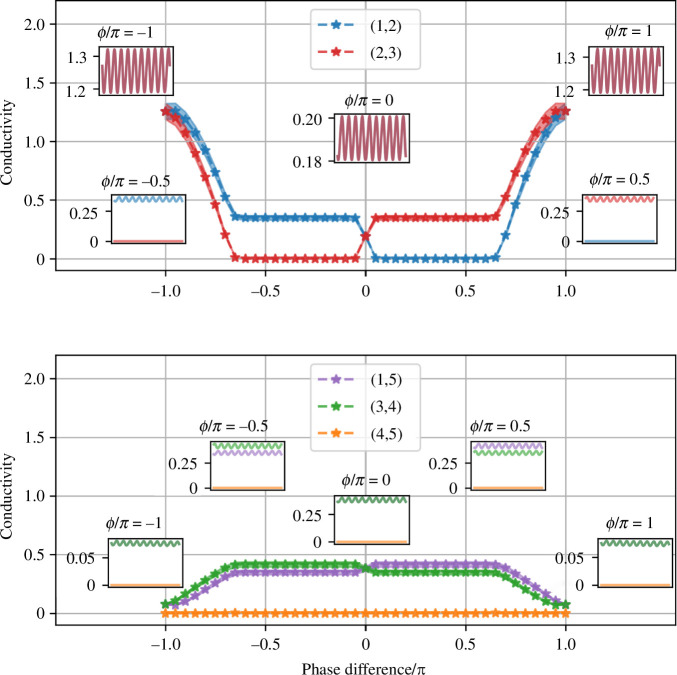
Bifurcation diagrams of the conductivity (thickness) of the edges on a $C_{5}$ graph, with the phase difference between the two oscillators as the bifurcation parameter. The *top *figure shows the conductivity of the edges on the shortest path, and the *bottom *figure shows the conductivity of the edges on the longest path. The parameters used are consistent with those in figure 6. Lines marked with asterisks represent median conductivity values, and shading denotes the amplitude of conductivity. Inserted subfigures show how the conductivity of edges changes as a function of time for different values of the phase difference.

In figure 7, we examine the change in conductivity as a function of the phase difference $\phi_{3}-\phi_{1}$, ranging from −π to π. When the phase difference is −π, the conductivity of the edges along the shortest path is at its maximum (see the points corresponding to a phase difference of −π at the leftmost part of figure 7). As the phase difference approaches one, the conductivity of the edges along the shortest path decreases, with the conductivity of the edge (2,3) approaching 0. Correspondingly, the conductivity of the edges (3,4) and (1,5) slowly increases. In this scenario, the two oscillating nodes are connected via the shortest path and have additional arms extending along the longer path, although these arms do not form a connection.

For phase differences between approximately −0.65π and 0, the network becomes disconnected: the conductivity of the edges remains constant and equals zero for the edge (1,2), as indicated in the corresponding region of figure 7. When the phase difference is 0, the two edges on the shortest path have identical conductivity values, as do the other two disconnected arms on the longer path. As the phase difference increases from 0 to approximately 0.65π, the conductivity of the edges is constant once more, with edge (2,3) now having zero conductivity. This pattern repeats, in the sense that the results are invariant under the relabelling of node 1 with 3, and node 4 with 5.

#### 3.2.2. Amplitude, frequency and phase differences

**Figure 8.**
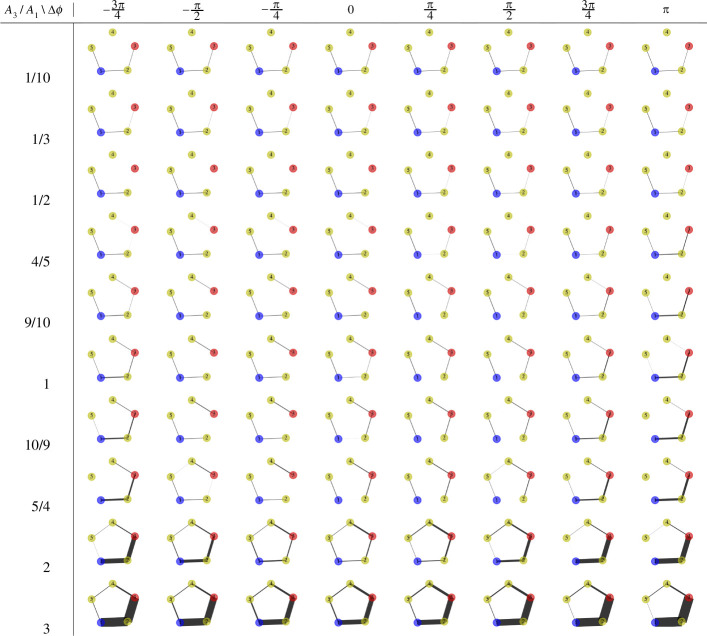
Conductivity results (median) obtained from the model on a $C_{5}$ graph over a sufficiently long time, subject to a changing amplitude $\alpha =A_{3}/A_{1}$ ($A_{1}=1$) and phase difference $\Delta \phi =\phi_{3}-\phi_{1}$ ($\phi_{1}=0$). Other parameters are consistent with those in figure 6. The thickness of the edges is proportional to the conductivity value.

When the amplitude or frequency of the two oscillatory nodes is not the same, the model exhibits a rich span of behaviours. In figure 8, we explore the resulting graphs as we vary both the phase difference $\Delta \phi =\phi_{3}-\phi_{1}$ and the amplitude ratio $A_{3}/A_{1}$. In these figures, the thickness of the edges indicates their conductivity after the simulation has run for a sufficient length of time. The middle row, where $A_{3}/A_{1}=1$, corresponds to the scenario presented in figure 7 and discussed in detail in the previous paragraph. Here, the graph transitions from initially being connected via the shortest path (Δϕ=−3π/4) with additional arms, to becoming disconnected (e.g. Δϕ=−π/4) and then to reconnecting (Δϕ=3π/4), with the majority of the flow following the shortest path (Δϕ=π).

When $A_{3}/A_{1}$ is close to 1, the results exhibit similar phase transitions to those seen when $A_{3}/A_{1}=1$; see the rows corresponding to Δϕ=9/10,10/9 in figure 8. As the amplitude ratio $A_{3}/A_{1}$ increases, edges generally exhibit higher conductivity values. For instance, when $A_{3}/A_{1}=2$, all edges display non-zero conductivity values for all possible phase differences Δϕ. Moreover, if Δϕ is close to −π or π, the edges along the shortest path exhibit higher conductivity values than others. With $A_{3}/A_{1}=3$ (and for larger ratios), the edges on the shortest path consistently show higher conductivity values than others, regardless of Δϕ. Thus, differences in amplitude can lead to a greater concentration of flow along the shorter path.

When the amplitude ratio $A_{3}/A_{1}$ is less than one, edges generally exhibit lower conductivity values. Specifically, when $A_{3}/A_{1}=1/2$, no edges incident on oscillatory node 3 have non-zero conductivity values when the phase difference Δϕ≤0. There is also only a weak connection between oscillatory node 3 and node 2 along the shortest path when Δϕ>0. When $A_{3}/A_{1}$ is 1/5 or 1/10, this results in a disconnected network for all possible values of Δϕ.

**Figure 9.**
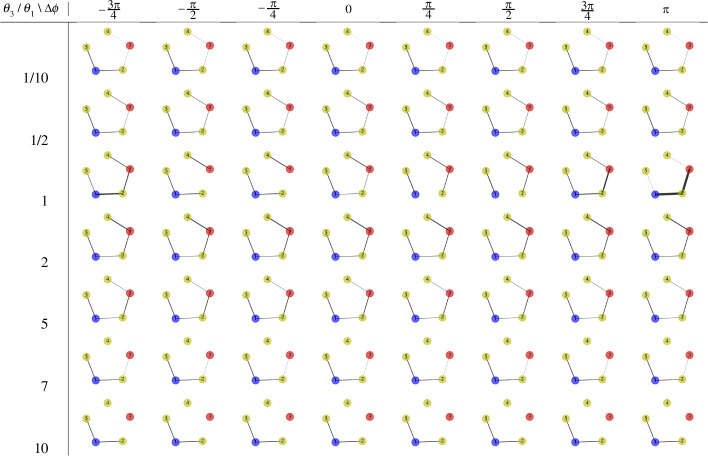
Conductivity results (median) obtained from the model on a $C_{5}$ graph over a sufficiently long time, subject to a changing frequency ratio $\gamma =\theta_{3}/\theta_{1}$ ($\theta_{1}=0.01$) and phase difference $\Delta \phi =\phi_{3}-\phi_{1}$ ($\phi_{1}=0$). Other parameters are consistent with those in figure 6. The thickness of the edges is proportional to the conductivity value.

In figure 9, we examine the resulting graphs as we change both phase difference $\Delta \phi =\phi_{3}-\phi_{1}$ and frequency ratio $\theta_{3}/\theta_{1}$. In these figures, the thickness of the edges, again, indicates their conductivity after the simulation has been run for a sufficiently long time. The third row where $\theta_{3}/\theta_{1}=1$ corresponds to the case presented in figure 8, exhibiting different features as the phase difference changes, as in the case of $A_{3}/A_{1}=1$ in the last paragraph.

However, when the frequency ratio $\theta_{3}/\theta_{1}\neq 1$, the resulting graphs remain the same at all phase difference values. As the frequency ratio $\theta_{3}/\theta_{1}$ increases, fewer edges have non-zero conductivity in general. For example, when $\theta_{3}/\theta_{1}=2$ or 5, only edges that are incident on either of the two oscillatory nodes have non-zero conductivity values. When $\theta_{3}/\theta_{1}=7$, the edge that is incident on oscillatory node 3 but not in the shortest path now has zero conductivity. In these two cases, the two oscillatory nodes are then connected via the shortest path and have additional arm(s). When $\theta_{3}/\theta_{1}=10$, no edges that are incident on oscillatory node 3 have non-zero conductivity, thus the graph becomes disconnected. Hence, the frequency can affect the exploration depth of the flow on the graph.

As the frequency ratio $\theta_{3}/\theta_{1}$ decreases, more edges have non-zero conductivity on the whole. Specifically, when $\theta_{3}/\theta_{1}=1/2$, all edges apart from the one that is not incident on either of the oscillatory nodes have non-zero conductivity. When $\theta_{3}/\theta_{1}=1/10$, all edges now have non-zero conductivity values, even though the conductivity of the one that has zero conductivity in the previous case is very small.

With all the above, we conclude that amplitude, frequency and phase all contain important information for the model to learn interesting patterns in the graph and the change in the environment.

### 3.3. Large graphs

We now proceed to examine the behaviour of the model, featuring oscillatory nodes, on larger graphs (see §2.5 for the set-up). The purpose of this investigation is to determine whether the model can reproduce large-scale patterns with shortest path connections between the nodes in the presence of external oscillations.

**Figure 10.**
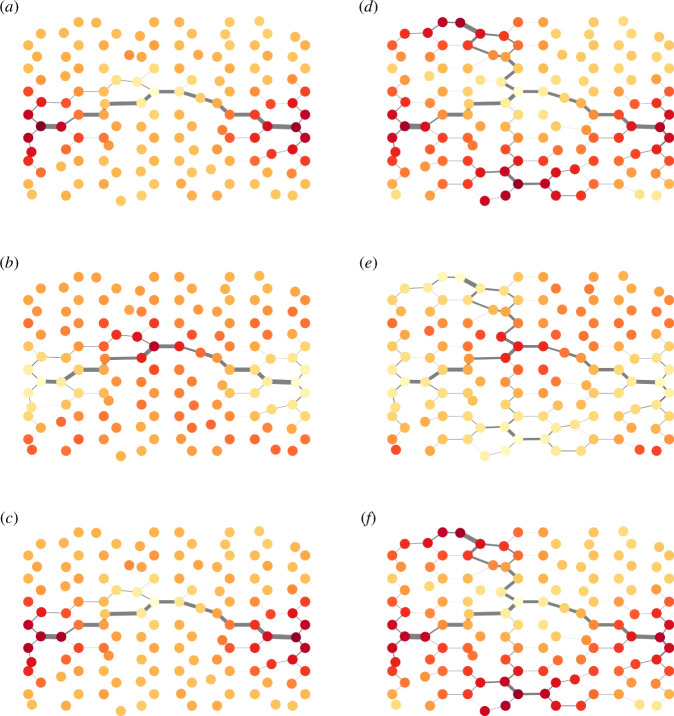
Snapshots of the graph at three different representative time points after a sufficiently long period, with different numbers of oscillatory nodes. Panels (*a*), (*b*) and (*c*) show snapshots of a graph with three oscillatory nodes, whereas panels (*d*), (*e*) and (*f*) show snapshots of a graph with five oscillatory nodes. The thickness of the edges indicates conductivity, and the colour of the nodes represents the number of particles at each node, with a redder colour indicating a higher number of particles.

In figure 10, we explore the behaviour of our model after a sufficiently long period in two different scenarios: one with three oscillatory nodes (the blue node in figure 2 and the two red nodes positioned horizontally in the middle of the figure) and another with five oscillatory nodes (one blue and four red). In both simulations, the flow primarily focuses on the shortest paths between the oscillatory nodes with the largest phase differences (illustrated by black connecting lines in figure 10). The oscillations dominate the particle behaviour. Tracing the sequence from figure 10*a* through 10*b* to 10*c*, we observe that the concentration of particles moves from the periphery to the centre and then back to the periphery again. Figure 10*d* through 10*e* to 10*f* demonstrates the same pattern but with five nodes.

While identifying parameter values that produce this outcome, we note that the frequency of oscillatory nodes plays a more significant role in large graphs than in the smaller graph examples previously examined. The oscillations should be sufficiently slow to allow information transfer from one oscillatory node to another before the particles return along their travelled paths. Simultaneously, the amplitude must be large enough to enable such communication before the conductivity decreases to zero. Therefore, in the simulations on larger graphs, we set the frequency to θ=0.001 and the amplitude to A=5 while keeping the reinforcement parameter at q=0.1 and the decay parameter at λ=0.01.

## 4. Discussion

Guided by Beer’s four steps [33,38], we have provided and investigated a mathematical framework inspired by organisms exhibiting basal cognition [13]. Under this framework, the formulation of the model is itself a contribution to this research area: we have built upon previous models of current reinforcement to explicitly include oscillations, demonstrating that such systems often exhibit the greatest flow on the shortest path between the oscillators.

We can think of our model as a demonstration of how organisms can monitor a dynamic environment, transporting particles (e.g. nutrients or signals) effectively between different locations. These abilities existed well before the development of nervous systems, let alone central nervous systems [13]. This makes our model a potential step towards a more realistic slime mould model [31], as it captures both synchronization and network construction observed in experiments of these organisms [23,42]. It also mimics other types of basal cognition, such as macromolecular networks in microbes [40] and signalling in fungi [39].

In a stationary environment, the current or flow of particles converges to an efficient transport network along the shortest path. Previous work on current-reinforced models has demonstrated that the shortest path is established in a model where the flow of particles is assumed to be in a steady state [43,44]. We have found that when explicitly modelling the dynamics of that flow, while the flow still concentrates on the shortest path, non-hyperbolic stable node configurations emerge. Specifically, non-hyperbolic stable nodes represent configurations where particles remain present on the non-optimal path, despite there being no flow in or out of that path. Unlike hyperbolic configurations, which exhibit strong attraction or repulsion, non-hyperbolic configurations feature slow dynamics, which means that particles ‘linger’ or are ‘stuck’ in suboptimal regions. There is a connection between these stuck particles and the concept of external memory in organisms with basal cognition. By leaving external cues in the environment, organisms can adapt their behaviour when environmental conditions change [60]. The particles on nodes corresponding to the longer path ’remember’ failed solutions and may be reactivated when conditions change.

The dynamic networks in our simulations produce both efficient flow and long-range oscillatory dynamics. While the nodes oscillate greatly in terms of number of particles at them, the connecting edges of the graph experience only small oscillations (this can be seen by contrasting the scale of the oscillations in conductivity and number of particles in figure 6). The resulting pattern has low-frequency responses to changes in formation over a long range, combined with a rapid flow of information across the system. We see this in figure 10, which is reminiscent of cross-frequency coupling, i.e. coupling between neurons of different frequencies, which has been proposed as a mechanism for working memory [6,7]. This is in contrast to the Kuramoto model, which captures synchronization but does not capture information transfer in the same way as our model. In our model, information transfer emerges from the interaction of coupled oscillators, which has also been observed experimentally in slime moulds, where the coupling of oscillatory dynamics allows for adaptive responses and efficient decision-making [61].

Comparing our model with recent slime mould models created by Alim *et al*. [52], our analysis reveals that current-based reinforcement coupled with oscillators can indeed find the shortest path, as suggested by Alim *et al*., and similar to what is found by Watanabe and Takamatsu [53]. However, in contrast to Watanabe and Takamatsu, the sum of the input and output rates in our model is not set to zero. Yet, after reaching a stable limit cycle, the input and output rates in our model cancel out, suggesting that the oscillators self-organize to have the total flux to and from the system balanced. Our work also aligns with Reid’s perspective on slime mould as a connected mass of oscillating units [62]. It further echoes early experiments by Takamatsu *et al*., displaying similar oscillatory behaviour in conductivity (i.e. thickness of the plasmodium network) in rings of slime mould oscillators [29,30].

Expanding our discussion to other species with basal cognition, we can draw parallels between our model and experiments in bacterial colonies that employ in-phase and anti-phase oscillatory behaviours during resource scarcity [35]. Similarly, the in-phase and out-of-phase oscillatory behaviours in our model are akin to the emergence of in-phase and out-of-phase oscillatory behaviours that are also found in plant shoots. For example, emergent maize plants grow in a group which exhibits synchronized oscillatory motions that may be in-phase or anti-phase [63]. Drawing connections to fungal mycelia, our model resonates with recent studies highlighting the bidirectional transport of signals and nutrients [39]. The flexibility of nodes to switch between acting as sources and sinks in our model provides a promising foundation for modelling bidirectional transportation within fungal mycelia [39].

Our analysis shows that not only phase but also amplitude and frequency of oscillations can induce the construction of efficient transport networks. This confirms the hypothesis (in a model) of Boussard *et al*., who suggested that all three variations could play a role in how slime moulds build networks [14]. Phase differences certainly play a relatively more important role, especially when the oscillatory nodes are close to being out of phase. In this case, the model nearly always directs almost all its flow along the shortest path between the nodes that are out of phase. However, any variation in phase, amplitude, frequency or a combination thereof, is sufficient to produce the basal cognition we observe in our simulations.

We do observe that very big frequency differences inhibit shortest-path formation. For example, the oscillating nodes become disconnected when the frequency ratio is 10 (see bottom row of figure 9). This, along with the need for the oscillators to be (somewhat) out of phase, gives some restrictions on the types of oscillations needed to create a flow between nodes. Amplitude primarily affects how far the particles can explore in the graph: too small amplitudes do not allow long-range communication. Our observations echo earlier studies on slime moulds, which show that frequency mismatches and phase relationships are crucial in coordinating oscillatory behaviour and directional movement in response to stimuli [64,65]. These studies demonstrate that when the frequency of external stimuli deviates significantly from the organism’s internal rhythm, it can disrupt coordinated movement, much like the disconnections observed in our model when frequency ratios are large.

Our model does not include feedback between oscillatory nodes, which we know are part of how living organisms assess, engage and adapt to the world around them. Such feedback could also potentially change the phase, amplitude and frequency of the oscillations towards values which better facilitate communications. Our next modelling steps would therefore be to incorporate feedback mechanisms for the oscillators into the model (as suggested in [14]). The introduction of feedback could involve the consideration of diverse forms of local information, $lc_{i}(t)$, such as the potential difference in particles ($\sum_{j\in \Gamma (i)}(N_{i}(t)-N_{j}(t))$), the absolute potential difference or a combination of potential difference and conductivity ($\sum_{j\in \Gamma (i)}(N_{i}(t)-N_{j}(t))D_{ij}(t)$). Moreover, an exploration could encompass various strategies for the phase transition, including the option of direct proportionality to local information ($\frac{d\phi_{i}(t)}{dt}=lc_{i}(t)$). An initial exploration using smaller networks could be undertaken to discern the impact of network topology on feedback forms, as done by Ma *et al*. [66]. Alternatively, an evolutionary adaptive approach, as advocated by Beer [38], offers another way to learn a feedback scheme.

In conclusion, the model we proposed here already has similar properties to many biological systems which exhibit basal (or even more complex forms of) cognition. We see it as a promising starting point for future simulation models of these phenomena.

## Figures and Tables

**Figure 11.**
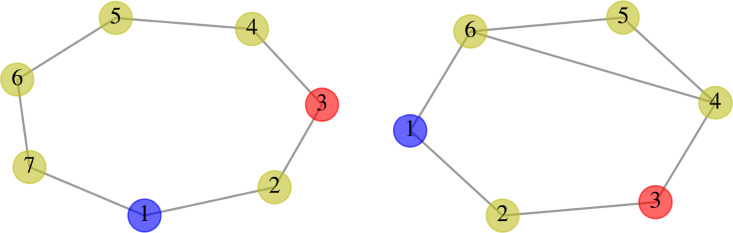
A $C_{7}$ graph (*left*) and a $C_{6}$ graph plus an extra edge between nodes 4 and 6 (*right*).

**Figure 12.**
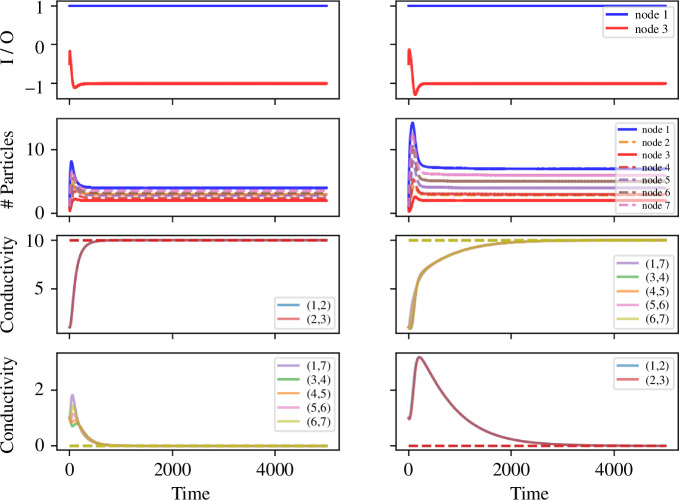
Results from the model on a $C_{7}$ graph, with node 1 as a source, node 3 as a sink, an inflow at rate a=1, an outflow at rate b=0.5, and the lengths of all edges are $l_{ij}=10$ apart from $l_{12}$. On the *left*, we set $l_{12}=10$, thus the path going through 1−7−6−5−4−3 is the shortest one. On the *right*, we set $l_{12}=50$ , thus the path going through 1-7-6-5-4-3 is the shortest one. The first row shows the input at node 1 and output at node 3, the second row plots the number of particles on each node, $N_{i}(t)$, the third row indicates the conductivity of the edges in the shortest path, and the last row indicates the conductivity of the remaining edges in the graph, where the dashed lines in the corresponding colour show the analytical results of the steady state.

**Figure 13.**
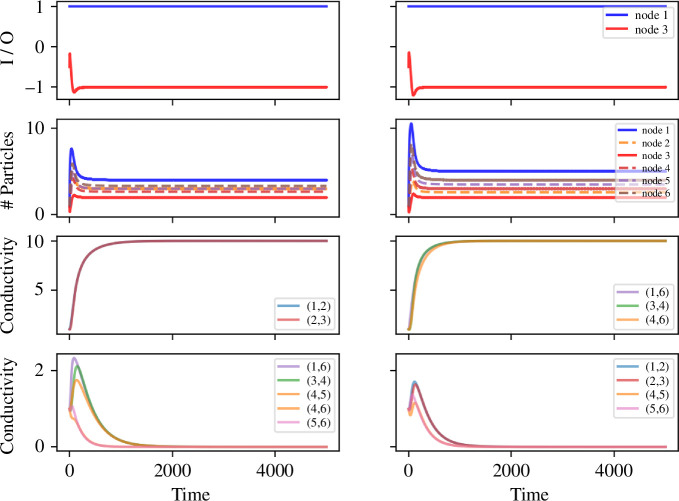
Results from the model on a $C_{6}$ graph plus an extra edge between nodes 4 and 6 (see the *right* graph in figure 11), with node 1 as a source, node 3 as a sink, an inflow at rate a=1, an outflow at rate b=0.5, and the lengths of all edges are $l_{ij}=10$ apart from $l_{12}$. On the *left*, we set $l_{12}=10$, thus the path going through 1−6−4−3 is the shortest one. On the *right*, we set $l_{12}=40$, thus the path going through 1-6-4-3 is the shortest one. The first row shows the input at node 1 and output at node 3, the second row plots the number of particles on each node, $N_{i}(t)$, the third row indicates the conductivity of the edges in the shortest path, and the last row indicates the conductivity of the remaining edges in the graph.

**Figure 14.**
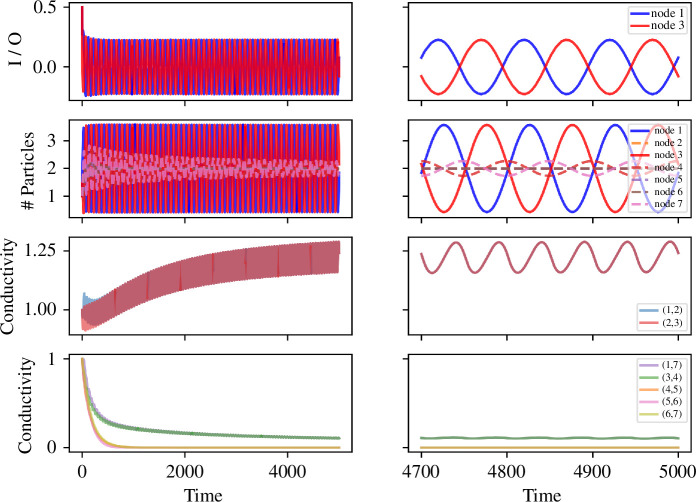
Results from the model on a $C_{7}$ graph, with nodes 1 and 3 as the oscillating nodes, rate b=0.5, amplitude $A_{1}=A_{3}=A=1$, frequency $\theta_{1}=\theta_{3}=\theta =0.01$, and phases $\phi_{1}=0$ and $\phi_{3}=\pi$, with the same reinforcement parameter q=0.1 and decay parameter λ=0.01 as in figure 3. The first row plots the actual input/output to the two nodes, the second row shows the number of particles in each node, the third row indicates the conductivity of the edges in the shortest path and the last row indicates the conductivity of the remaining edges in the graph, with the full profile on the *left* and the change in the long term on the *right*.

**Figure 15.**
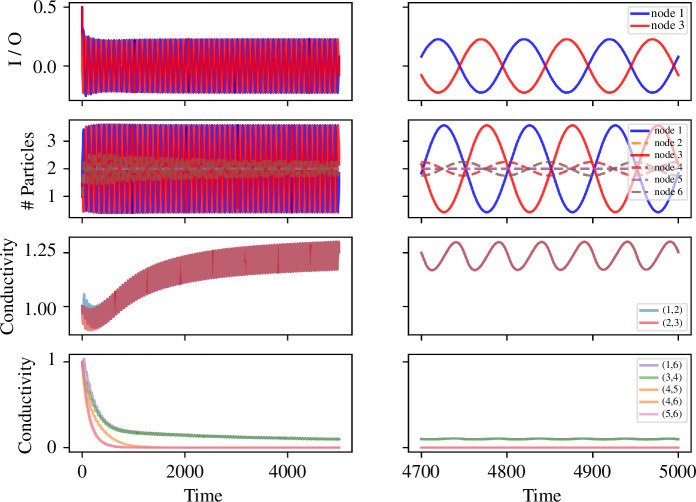
Results from the model on a $C_{6}$ graph plus an extra edge between nodes 4 and 6 (the *right* in figure 11), with nodes 1 and 3 as the oscillating nodes, rate b=0.5, amplitude $A_{1}=A_{3}=A=1$, frequency $\theta_{1}=\theta_{3}=\theta =0.01$, and phases $\phi_{1}=0$ and $\phi_{3}=\pi$, with the same reinforcement parameter q=0.1 and decay parameter λ=0.01 as in figure 3. The first row plots the actual input/output to the two nodes, the second row shows the number of particles in each node, the third row indicates the conductivity of the edges in the shortest path and the last row indicates the conductivity of the remaining edges in the graph, with the full profile on the *left* and the change in the long term on the *right*.

## Data Availability

The code used to generate the simulations presented in this paper is openly accessible on Zenodo at [67].

## Appendix A. Non-oscillatory sources and sinks

### A.1. Steady states

This section of the appendix derives the results for the steady states as presented in §3.1. The equilibrium points of the system are determined by the solutions to the following set of equations:

(A 1)
$$\begin{array}{lrlr} & & a+\frac{\left(N_{2}^{*}-N_{1}^{*}\right)}{l_{12}}D_{12}^{*}+\frac{\left(N_{5}^{*}-N_{1}^{*}\right)}{l_{51}}D_{51}=0, & \end{array}$$

(A 2)
$$\begin{array}{lrlr} & & \frac{\left(N_{1}^{*}-N_{2}^{*}\right)}{l_{12}}D_{12}^{*}+\frac{\left(N_{3}^{*}-N_{2}^{*}\right)}{l_{23}}D_{23}^{*}=0, & \end{array}$$

(A 3)
$$\begin{array}{lrlr} & & -bN_{3}^{*}+\frac{\left(N_{2}^{*}-N_{3}^{*}\right)}{l_{23}}D_{23}^{*}+\frac{\left(N_{4}^{*}-N_{3}^{*}\right)}{l_{34}}D_{34}^{*}=0, & \end{array}$$

(A 4)
$$\begin{array}{lrlr} & & \frac{\left(N_{3}^{*}-N_{4}^{*}\right)}{l_{34}}D_{34}^{*}+\frac{\left(N_{5}^{*}-N_{4}^{*}\right)}{l_{45}}D_{45}^{*}=0, & \end{array}$$

(A 5)
$$\begin{array}{lrlr} & & \frac{\left(N_{1}^{*}-N_{5}^{*}\right)}{l_{51}}D_{51}^{*}+\frac{\left(N_{4}^{*}-N_{5}^{*}\right)}{l_{45}}D_{45}^{*}=0, & \end{array}$$

(A 6)
$$\begin{array}{lrlr} & & q\frac{\left|N_{1}^{*}-N_{2}^{*}\right|}{l_{12}}D_{12}^{*}-\lambda D_{12}^{*}=0, & \end{array}$$

(A 7)
$$\begin{array}{lrlr} & & q\frac{\left|N_{2}^{*}-N_{3}^{*}\right|}{l_{23}}D_{23}^{*}-\lambda D_{23}^{*}=0, & \end{array}$$

(A 8)
$$\begin{array}{lrlr} & & q\frac{\left|N_{3}^{*}-N_{4}^{*}\right|}{l_{34}}D_{34}^{*}-\lambda D_{34}^{*}=0, & \end{array}$$

(A 9)
$$\begin{array}{lrlr} & & q\frac{\left|N_{4}^{*}-N_{5}^{*}\right|}{l_{45}}D_{45}^{*}-\lambda D_{45}^{*}=0, & \end{array}$$

(A 10)
$$\begin{array}{lrlr} & & q\frac{\left|N_{5}^{*}-N_{1}^{*}\right|}{l_{51}}D_{51}^{*}-\lambda D_{51}^{*}=0. & \end{array}$$

In general, the steady states for the conductivity are given by the equation

(A 11)
$$\begin{array}{lrr} & q\frac{\left|N_{i}^{*}-N_{j}^{*}\right|}{l_{ij}}D_{ij}^{*}-\lambda D_{ij}^{*}=0. & \end{array}$$

For this to hold, either $D_{ij}^{*}=0$ or $\left|N_{i}^{*}-N_{j}^{*}\right|=\frac{\lambda l_{ij}}{q}$. If $D_{ij}^{*}\neq 0$, then $\left|N_{i}^{*}-N_{j}^{*}\right|=\frac{\lambda l_{ij}}{q}$, and thus, equation (A 11)[Disp-formula A1-E11] can be simplified to

(A 12)
$$\begin{array}{lcr} & \pm \frac{\lambda }{q}D_{i-1,i}^{*}+\pm \frac{\lambda }{q}D_{i,i+1}^{*}=0. & \end{array}$$

As $D_{ij}(t)$ denotes the conductivity, we know that $D_{ij}(t)\geq 0$ and thus, the two addends must have opposite signs. This gives the solution

(A 13)
$$\begin{array}{lcr} & D_{i-1,i}^{*}=D_{i,i+1}^{*}. & \end{array}$$

This also implies that for a node i, which is neither a source nor a sink, $\left(N_{i-1}^{*}-N_{i}^{*}\right)$ and $\left(N_{i+1}-N_{i}\right)$ must have opposite signs. We can now consider the following cases:

**Case 1:** $D_{51}^{*}=0\;and\;D_{12}^{*}\neq 0$

If $D_{51}^{*}$ is zero and $D_{12}^{*}$ is non-zero, then $\left|N_{2}^{*}-N_{1}^{*}\right|=\frac{\lambda l_{12}}{q}$. We thus have that

(A 14)
$$\begin{array}{lcr} & a\pm \frac{\lambda l_{12}}{l_{12}q}D_{12}^{*}=0. & \end{array}$$

We know that the conductivity must be positive, so

(A 15)
$$\begin{array}{lcr} & D_{12}^{*}=\frac{aq}{\lambda }, & \end{array}$$

as a, q and λ are positive. This means that

(A 16)
$$\begin{array}{lcr} & (N_{2}^{*}-N_{1}^{*})=-\frac{\lambda l_{12}}{q}. & \end{array}$$

From [Disp-formula A1-E13]equation (A 13) we know that $D_{12}^{*}=D_{23}^{*}$ and $D_{34}^{*}=D_{45}^{*}=D_{51}^{*}$, so the steady states for the conductivity will in this case be given by $(D_{12}^{*},D_{23}^{*},D_{34}^{*},D_{45}^{*},D_{51}^{*})=\left(\frac{aq}{\lambda },\frac{aq}{\lambda },0,0,0\right)$.

Now consider the equation for the change of particles at node with a sink,

(A 17)
$$\frac{dN_{3}(t)}{dt}=-bN_{3}+\frac{\left(N_{2}-N_{3}\right)}{l_{23}}D_{23}+\frac{\left(N_{4}-N_{3}\right)}{l_{34}}D_{34}.$$

The steady states will be given the solution to the equation

(A 18)
$$-bN_{3}^{*}+\frac{\left(N_{2}^{*}-N_{3}^{*}\right)}{l_{23}}D_{23}^{*}+\frac{\left(N_{4}^{*}-N_{3}^{*}\right)}{l_{34}}D_{34}^{*}=0.$$

Now, if $(D_{12}^{*},D_{23}^{*},D_{34}^{*},D_{45}^{*},D_{51}^{*})=\left(\frac{aq}{\lambda },\frac{aq}{\lambda },0,0,0\right)$, then the equation can be simplified to

(A 19)
$$\begin{array}{lcr} & -bN_{3}^{*}+\frac{\left(N_{2}^{*}-N_{3}^{*}\right)}{l_{23}}D_{23}^{*}=0, & \end{array}$$

where $N_{2}^{*}-N_{3}^{*}=\pm \frac{\lambda l_{23}}{q}$ and $D_{23}^{*}=\frac{aq}{\lambda }$, which gives the following:

(A 20)
$$\begin{array}{lcr} & -bN_{3}^{*}\pm \frac{\frac{\lambda l_{23}}{q}}{l_{23}}\frac{aq}{\lambda }=0. & \end{array}$$

As $N_{3}$ denotes the number of particles at node 3, $N_{3}$ must be positive and we get

(A 21)
$$\begin{array}{lcr} & N_{3}^{*}=\frac{a}{b}. & \end{array}$$

This implies that $N_{2}^{*}-N_{3}^{*}=+\frac{\lambda l_{23}}{q}$, and thus

(A 22)
$$\begin{array}{lcr} & N_{2}^{*}=\frac{a}{b}+\frac{\lambda l_{23}}{q}. & \end{array}$$

We know that $(N_{2}^{*}-N_{1}^{*})=-\frac{\lambda l_{12}}{q}$, so

(A 23)
$$\begin{array}{lcr} & N_{1}^{*}=\frac{a}{b}+\frac{\lambda l_{23}}{q}+\frac{\lambda l_{12}}{q}. & \end{array}$$

There are no exact solutions for $N_{4}^{*}$ and $N_{5}^{*}$, so for case 1, we have the following steady state:

$$\begin{array}{r} (N_{1}^{*},N_{2}^{*},N_{3}^{*},N_{4}^{*},N_{5}^{*},D_{12}^{*},D_{23}^{*},D_{34}^{*},D_{45}^{*},D_{51}^{*})=\\ \frac{a}{b}+\frac{\lambda l_{23}}{q}+\frac{\lambda l_{12}}{q},\frac{a}{b}+\frac{\lambda l_{23}}{q},\frac{a}{b},N_{4}^{*},N_{5}^{*},\frac{aq}{\lambda },\frac{aq}{\lambda },0,0,0). \end{array}$$

**Case 2**: $D_{12}^{*}=0$ and $D_{51}^{*}\neq 0$

If $D_{12}^{*}$ is zero and $D_{51}^{*}$ is non-zero, then $\left|N_{5}^{*}-N_{1}^{*}\right|=\frac{\lambda l_{51}}{q}$, and thus

(A 24)
$$\begin{array}{lcr} & D_{51}^{*}=\frac{aq}{\lambda }, & \end{array}$$

and

(A 25)
$$\begin{array}{lcr} & N_{5}^{*}-N_{1}^{*}=-\frac{\lambda l_{51}}{q}. & \end{array}$$

Again, consider the equation for the change of particles at node with a sink,

(A 26)
$$\frac{dN_{3}(t)}{dt}=-bN_{3}+\frac{\left(N_{2}-N_{3}\right)}{l_{23}}D_{23}+\frac{\left(N_{4}-N_{3}\right)}{l_{34}}D_{34}.\;$$

The steady states will be given the solution to the equation

(A 27)
$$-bN_{3}^{*}+\frac{\left(N_{2}^{*}-N_{3}^{*}\right)}{l_{23}}D_{23}^{*}+\frac{\left(N_{4}^{*}-N_{3}^{*}\right)}{l_{34}}D_{34}^{*}=0.\;$$

Now, if $(D_{12}^{*},D_{23}^{*},D_{34}^{*},D_{45}^{*},D_{51}^{*})=\left(0,0,\frac{aq}{\lambda },\frac{aq}{\lambda },\frac{aq}{\lambda }\right)$, then the equation can be simplified to

(A 28)
$$\begin{array}{lcr} & -bN_{3}^{*}+\frac{\left(N_{4}^{*}-N_{3}^{*}\right)}{l_{34}}D_{34}^{*}=0, & \end{array}$$

where $N_{4}^{*}-N_{3}^{*}=\pm \frac{\lambda l_{34}}{q}$ and $D_{34}^{*}=\frac{aq}{\lambda }$, which gives the following:

(A 29)
$$\begin{array}{lcr} & -bN_{3}^{*}\pm \frac{\frac{\lambda l_{34}}{q}}{l_{34}}\frac{aq}{\lambda }=0. & \end{array}$$

As $N_{3}$ denotes the number of particles at node 3, $N_{3}$ must be positive and thus $N_{4}^{*}-N_{3}^{*}=+\frac{\lambda l_{34}}{q}$. Hence, we get

(A 30)
$$\begin{array}{lcr} & N_{3}^{*}=\frac{a}{b}. & \end{array}$$

We know that $N_{4}^{*}-N_{3}^{*}=\frac{\lambda l_{34}}{q}$, so

(A 31)
$$\begin{array}{lcr} & N_{4}^{*}=\frac{a}{b}+\frac{\lambda l_{34}}{q}. & \end{array}$$

As node 4 is neither a source, nor sink, we know that $N_{4}^{*}-N_{5}^{*}$ and $N_{4}^{*}-N_{3}^{*}$ have opposite signs. As $N_{4}^{*}-N_{3}^{*}$ is positive, this implies that

(A 32)
$$\begin{array}{lcr} & N_{4}^{*}-N_{5}^{*}=-\frac{\lambda l_{45}}{q}, & \end{array}$$

and thus

(A 33)
$$\begin{array}{lcr} & N_{5}^{*}=\frac{a}{b}+\frac{\lambda l_{34}}{q}+\frac{\lambda l_{45}}{q}. & \end{array}$$

As $N_{4}^{*}-N_{5}^{*}$ is negative, $N_{1}^{*}-N_{5}^{*}$ must be positive and thus

(A 34)
$$\begin{array}{lcr} & N_{5}^{*}-N_{1}^{*}=\frac{\lambda l_{51}}{q}, & \end{array}$$

which gives

(A 35)
$$\begin{array}{lcr} & N_{1}^{*}-\left(\frac{a}{b}+\frac{\lambda l_{34}}{q}+\frac{\lambda l_{45}}{q}\right)=\frac{\lambda }{l_{51}}q, & \end{array}$$

so

(A 36)
$$\begin{array}{lcr} & N_{1}^{*}=\frac{a}{b}+\frac{\lambda l_{34}}{q}+\frac{\lambda l_{45}}{q}+\frac{\lambda l_{51}}{q}. & \end{array}$$

As for $N_{4}^{*}$ and $N_{5}^{*}$ in case 1, there are no exact solutions for $N_{2}^{*}$ in case 2. Thus the steady states are given by

$$\begin{array}{r} (N_{1}^{*},N_{2}^{*},N_{3}^{*},N_{4}^{*},N_{5}^{*},D_{12}^{*},D_{23}^{*},D_{34}^{*},D_{45}^{*},D_{51}^{*})=\\ (\frac{a}{b}+\frac{\lambda l_{34}}{q}+\frac{\lambda l_{45}}{q}+\frac{\lambda l_{51}}{q},N_{2}^{*},\frac{a}{b},\frac{a}{b}+\frac{\lambda l_{34}}{q},\frac{a}{b}+\frac{\lambda l_{34}}{q}+\frac{\lambda l_{45}}{q},0,0,\frac{aq}{\lambda },\frac{aq}{\lambda },\frac{aq}{\lambda }). \end{array}$$

**Case 3:** $D_{12}^{*}\neq 0$ and $D_{51}^{*}\neq 0$

Now, if neither $D_{12}$ nor $D_{51}$ is non-zero we have that

(A 37)
$$\begin{array}{lcr} & a\pm \frac{\lambda }{q}D_{12}^{*}\pm \frac{\lambda }{q}D_{51}^{*}=0, & \end{array}$$

which gives

(A 38)
$$\begin{array}{lcr} & \pm D_{12}^{*}\pm D_{51}^{*}=\frac{aq}{\lambda }. & \end{array}$$

As the conductivity is the same on the edges going from a non-source and non-sink, we know that

(A 39)
$$\begin{array}{lcr} & D_{12}^{*}=D_{23}^{*}, & \end{array}$$

and

(A 40)
$$\begin{array}{lcr} & D_{34}^{*}=D_{45}^{*}=D_{51}^{*}. & \end{array}$$

If we assume that the conductivity is the same on all edges, we get that

(A 41)
$$\begin{array}{lcr} & a\pm \frac{\lambda }{q}D_{12}^{*}\pm \frac{\lambda }{q}D_{12}^{*}=0. & \end{array}$$

As the conductivity must be positive, we have

(A 42)
$$\begin{array}{lcr} & a-\frac{\lambda }{q}D_{12}^{*}-\frac{\lambda }{q}D_{12}^{*}=0, & \end{array}$$

and thus

(A 43)
$$\begin{array}{lcr} & D_{12}^{*}=\frac{aq}{2\lambda }. & \end{array}$$

Hence,

(A 44)
$$\begin{array}{lcr} & D_{12}^{*}=D_{23}^{*}=D_{34}^{*}=D_{45}^{*}=D_{51}^{*}=\frac{aq}{2\lambda }. & \end{array}$$

This also means that

(A 45)
$$\begin{array}{lcr} & (N_{2}^{*}-N_{1}^{*})=-\frac{\lambda l_{12}}{q}, & \end{array}$$

and

(A 46)
$$\begin{array}{lcr} & (N_{5}^{*}-N_{1}^{*})=-\frac{\lambda l_{51}}{q}. & \end{array}$$

Now using that for a node, i, which is neither a source nor a sink, $\left(N_{i-1}^{*}-N_{i}^{*}\right)$ and $\left(N_{i+1}^{*}-N_{i}^{*}\right)$ must have opposite signs, we know that

(A 47)
$$\begin{array}{lrr} & (N_{3}^{*}-N_{2}^{*})=-\frac{\lambda l_{23}}{q}, & \end{array}$$

(A 48)
$$\begin{array}{lrr} & (N_{4}^{*}-N_{5}^{*})=-\frac{\lambda l_{45}}{q}, & \end{array}$$

(A 49)
$$\begin{array}{lrr} & (N_{3}^{*}-N_{4}^{*})=-\frac{\lambda l_{34}}{q}. & \end{array}$$

To find the steady states of the number of particles, we first look at the following equation:

(A 50)
$$\begin{array}{lcr} & -bN_{3}^{*}+\frac{\left(N_{2}^{*}-N_{3}^{*}\right)}{l_{23}}D_{23}^{*}+\frac{\left(N_{4}^{*}-N_{3}^{*}\right)}{l_{34}}D_{34}^{*}=0. & \end{array}$$

Inserting $(N_{2}^{*}-N_{3}^{*})=\frac{\lambda l_{23}}{q}$, $(N_{4}^{*}-N_{3}^{*})=\frac{\lambda l_{34}}{q}$, and $D_{23}^{*}=D_{34}^{*}=\frac{aq}{2\lambda }$, we obtain

(A 51)
$$\begin{array}{lcr} & -bN_{3}^{*}+\frac{a}{2}+\frac{a}{2}=0, & \end{array}$$

and thus

(A 52)
$$\begin{array}{lcr} & N_{3}^{*}=\frac{a}{b}. & \end{array}$$

This means that

(A 53)
$$\begin{array}{lcr} & N_{4}^{*}=\frac{a}{b}+\frac{\lambda l_{34}}{q}, & \end{array}$$

(A 54)
$$\begin{array}{lcr} & N_{2}^{*}=\frac{a}{b}+\frac{\lambda l_{23}}{q}, & \end{array}$$

(A 55)
$$\begin{array}{lcr} & N_{1}^{*}=\frac{a}{b}+\frac{\lambda l_{23}}{q}+\frac{\lambda l_{12}}{q}, & \end{array}$$

and

(A 56)
$$\begin{array}{lcr} & N_{5}^{*}=\frac{a}{b}+\frac{\lambda l_{34}}{q}+\frac{\lambda l_{45}}{q}. & \end{array}$$

But we also know that $(N_{5}^{*}-N_{1}^{*})=-\frac{\lambda l_{51}}{q}$, so combining equations (A 55) and (A 56) we get

(A 57)
$$\begin{array}{lcr} & N_{5}^{*}-N_{1}^{*}=\frac{a}{b}+\frac{\lambda l_{34}}{q}+\frac{\lambda l_{45}}{q}-\frac{a}{b}+\frac{\lambda l_{23}}{q}+\frac{\lambda l_{12}}{q}=-\frac{\lambda l_{51}}{q}, & \end{array}$$

which implies that

(A 58)
$$\begin{array}{lcr} & l_{12}+l_{23}=l_{34}+l_{45}+l_{51}. & \end{array}$$

Thus for the steady states to be

$$\begin{array}{r} (N_{1}^{*},N_{2}^{*},N_{3}^{*},N_{4}^{*},N_{5}^{*},D_{12}^{*},D_{23}^{*},D_{34}^{*},D_{45}^{*},D_{51}^{*})=\\ (\frac{a}{b}+\frac{\lambda l_{23}}{q}+\frac{\lambda l_{12}}{q},\frac{a}{b}+\frac{\lambda l_{23}}{q},\frac{a}{b},\frac{a}{b}+\frac{\lambda l_{34}}{q},\frac{a}{b}+\frac{\lambda l_{34}}{q}+\frac{\lambda l_{45}}{q},\frac{aq}{2\lambda },\frac{aq}{2\lambda },\frac{aq}{2\lambda },\frac{aq}{2\lambda },\frac{aq}{2\lambda }), \end{array}$$

the different paths between the source and the sink must have the same total length.

### A.2. Stability

As an initial step to investigate the stability of the system described by equation (3.1), we analyse the Jacobian matrix, denoted as J. The expression for J is given by the following extensive matrix:

(A 59)
$$\begin{array}{r} J=\left(\begin{array}{cccccccccc} -\frac{D_{12}}{l_{12}}-\frac{D_{51}}{l_{51}} & \frac{D_{12}}{l_{12}} & 0 & 0 & \frac{D_{51}}{l_{51}} & \frac{N_{2}-N_{1}}{l_{12}} & 0 & 0 & 0 & \frac{N_{5}-N_{1}}{l_{51}}\\ \frac{D_{12}}{l_{12}} & -\frac{D_{12}}{l_{12}}-\frac{D_{23}}{l_{23}} & \frac{D_{23}}{l_{23}} & 0 & 0 & \frac{N_{2}-N_{1}}{l_{12}} & \frac{N_{3}-N_{2}}{l_{23}} & 0 & 0 & 0\\ 0 & \frac{D_{23}}{l_{23}} & -b-\frac{D_{23}}{l_{23}}-\frac{D_{34}}{l_{34}} & \frac{D_{34}}{l_{34}} & 0 & 0 & \frac{N_{3}-N_{2}}{l_{23}} & \frac{N_{4}-N_{3}}{l_{34}} & 0 & 0\\ 0 & 0 & \frac{D_{34}}{l_{34}} & -\frac{D_{34}}{l_{34}}-\frac{D_{45}}{l_{45}} & \frac{D_{45}}{l_{45}} & 0 & 0 & \frac{N_{3}-N_{4}}{l_{34}} & \frac{N_{5}-N_{4}}{l_{45}} & 0\\ \frac{D_{51}}{l_{51}} & 0 & 0 & \frac{D_{45}}{l_{45}} & -\frac{D_{45}}{l_{45}}-\frac{D_{51}}{l_{51}} & 0 & 0 & 0 & \frac{N_{4}-N_{5}}{l_{45}} & \frac{N_{1}-N_{5}}{l_{51}}\\ \frac{qsgn\left(N_{1}-N_{2}\right)D_{12}}{l_{12}} & \frac{qsgn\left(N_{1}-N_{2}\right)D_{12}}{l_{12}} & 0 & 0 & 0 & \frac{q|N_{1}-N_{2}|}{l_{12}}-\lambda & 0 & 0 & 0 & 0\\ 0 & \frac{qsgn\left(N_{2}-N_{3}\right)D_{23}}{l_{23}} & \frac{qsgn\left(N_{2}-N_{3}\right)D_{23}}{l_{23}} & 0 & 0 & 0 & \frac{q|N_{2}-N_{3}|}{l_{23}}-\lambda & 0 & 0 & 0\\ 0 & 0 & \frac{qsgn\left(N_{3}-N_{4}\right)D_{34}}{l_{34}} & \frac{qsgn\left(N_{3}-N_{4}\right)D_{34}}{l_{34}} & 0 & 0 & 0 & \frac{q|N_{3}-N_{4}|}{l_{34}}-\lambda & 0 & 0\\ 0 & 0 & 0 & \frac{qsgn\left(N_{4}-N_{5}\right)D_{45}}{l_{45}} & \frac{qsgn\left(N_{4}-N_{5}\right)D_{45}}{l_{45}} & 0 & 0 & 0 & \frac{q|N_{4}-N_{5}|}{l_{45}}-\lambda & 0\\ \frac{qsgn\left(N_{5}-N_{1}\right)D_{51}}{l_{51}} & 0 & 0 & 0 & \frac{qsgn\left(N_{5}-N_{1}\right)D_{51}}{l_{51}} & 0 & 0 & 0 & 0 & \frac{q|N_{5}-N_{1}|}{l_{51}}-\lambda \end{array}\right). \end{array}$$

Upon evaluating J at equilibrium $E_{1}$, the resulting matrix $J_{E_{1}}$ is given by

(A 60)
$$J_{E_{1}}=\left(\begin{array}{cccccccccc} -\frac{a\;q}{l_{12}\;\lambda } & \frac{a\;q}{l_{12}\;\lambda } & 0 & 0 & 0 & -\frac{\lambda }{q} & 0 & 0 & 0 & -\frac{\frac{a}{b}-N_{5}+\frac{l_{12}\;\lambda }{q}+\frac{l_{23}\;\lambda }{q}}{l_{51}}\\ \frac{a\;q}{l_{12}\;\lambda } & -\frac{a\;l_{23}\;q}{\lambda }-\frac{a\;q}{l_{12}\;\lambda } & \frac{a\;l_{23}\;q}{\lambda } & 0 & 0 & \frac{\lambda }{q} & -\frac{{l_{23}}^{2}\;\lambda }{q} & 0 & 0 & 0\\ 0 & \frac{a\;q}{l_{23}\;\lambda } & -b-\frac{a\;q}{l_{23}\;\lambda } & 0 & 0 & 0 & \frac{\lambda }{q} & \frac{N_{4}-\frac{a}{b}}{l_{34}} & 0 & 0\\ 0 & 0 & 0 & 0 & 0 & 0 & 0 & -\frac{N_{4}-\frac{a}{b}}{l_{34}} & -l_{45}\;\left(N_{4}-N_{5}\right) & 0\\ 0 & 0 & 0 & 0 & 0 & 0 & 0 & 0 & l_{45}\;\left(N_{4}-N_{5}\right) & \frac{\frac{a}{b}-N_{5}+\frac{l_{12}\;\lambda }{q}+\frac{l_{23}\;\lambda }{q}}{l_{51}}\\ \frac{a\;q^{2}\;}{l_{12}\;\lambda } & -\frac{a\;q^{2}\;}{l_{12}\;\lambda } & 0 & 0 & 0 & 0 & 0 & 0 & 0 & 0\\ 0 & \frac{a\;q^{2}\;}{l_{23}\;\lambda } & -\frac{a\;q^{2}\;}{l_{23}\;\lambda } & 0 & 0 & 0 & 0 & 0 & 0 & 0\\ 0 & 0 & 0 & 0 & 0 & 0 & 0 & \frac{q\;\left|N_{4}-\frac{a}{b}\right|}{l_{34}}-\lambda & 0 & 0\\ 0 & 0 & 0 & 0 & 0 & 0 & 0 & 0 & \frac{q\;\left|N_{4}-N_{5}\right|}{l_{45}}-\lambda & 0\\ 0 & 0 & 0 & 0 & 0 & 0 & 0 & 0 & 0 & \frac{q\;\left|\frac{a}{b}-N_{5}+\frac{l_{12}\;\lambda }{q}+\frac{l_{23}\;\lambda }{q}\right|}{l_{51}}-\lambda \end{array}\right).$$

As we have two zero columns, there will be two zero eigenvalues, and thus the determinant will also be zero, meaning that the equilibrium is non-hyperbolic. The two eigenvectors corresponding to the two zero eigenvalues will be $\left(0,0,0,N_{4}^{*},0,0,0,0,0,0\right)$ and $\left(0,0,0,0,N_{5}^{*},0,0,0,0,0\right)$.

Evaluating J at $E_{2}$ we obtain

(A 61)
$$J_{E_{2}}=\left(\begin{array}{cccccccccc} -\frac{a\;q}{l_{51}\;\lambda } & 0 & 0 & 0 & \frac{a\;q}{l_{51}\;\lambda } & -\frac{\frac{a}{b}-N_{2}+\frac{l_{34}\;\lambda }{q}+\frac{l_{45}\;\lambda }{q}+\frac{l_{51}\;\lambda }{q}}{l_{12}} & 0 & 0 & 0 & -\frac{\lambda }{q}\\ 0 & 0 & 0 & 0 & 0 & \frac{\frac{a}{b}-N_{2}+\frac{l_{34}\;\lambda }{q}+\frac{l_{45}\;\lambda }{q}+\frac{l_{51}\;\lambda }{q}}{l_{12}} & -l_{23}\;\left(N_{2}-\frac{a}{b}\right) & 0 & 0 & 0\\ 0 & 0 & -b-\frac{a\;q}{l_{34}\;\lambda } & \frac{a\;q}{l_{34}\;\lambda } & 0 & 0 & \frac{N_{2}-\frac{a}{b}}{l_{23}} & \frac{\lambda }{q} & 0 & 0\\ 0 & 0 & \frac{a\;q}{l_{34}\;\lambda } & -\frac{a\;l_{45}\;q}{\lambda }-\frac{a\;q}{l_{34}\;\lambda } & \frac{a\;l_{45}\;q}{\lambda } & 0 & 0 & -\frac{\lambda }{q} & \frac{{l_{45}}^{2}\;\lambda }{q} & 0\\ \frac{a\;q}{l_{51}\;\lambda } & 0 & 0 & \frac{a\;l_{45}\;q}{\lambda } & -\frac{a\;l_{45}\;q}{\lambda }-\frac{a\;q}{l_{51}\;\lambda } & 0 & 0 & 0 & -\frac{{l_{45}}^{2}\;\lambda }{q} & \frac{\lambda }{q}\\ 0 & 0 & 0 & 0 & 0 & \frac{q\;\left|\frac{a}{b}-N_{2}+\frac{l_{34}\;\lambda }{q}+\frac{l_{45}\;\lambda }{q}+\frac{l_{51}\;\lambda }{q}\right|}{l_{12}}-\lambda & 0 & 0 & 0 & 0\\ 0 & 0 & 0 & 0 & 0 & 0 & \frac{q\;\left|N_{2}-\frac{a}{b}\right|}{l_{23}}-\lambda & 0 & 0 & 0\\ 0 & 0 & -\frac{a\;q^{2}\;sgn\left(\frac{l_{34}\;\lambda }{q}\right)}{l_{34}\;\lambda } & \frac{a\;q^{2}\;sgn\left(\frac{l_{34}\;\lambda }{q}\right)}{l_{34}\;\lambda } & 0 & 0 & 0 & 0 & 0 & 0\\ 0 & 0 & 0 & -\frac{a\;q^{2}\;sgn\left(\frac{l_{45}\;\lambda }{q}\right)}{l_{45}\;\lambda } & \frac{a\;q^{2}\;sgn\left(\frac{l_{45}\;\lambda }{q}\right)}{l_{45}\;\lambda } & 0 & 0 & 0 & 0 & 0\\ \frac{a\;q^{2}\;sgn\left(\frac{l_{51}\;\lambda }{q}\right)}{l_{51}\;\lambda } & 0 & 0 & 0 & -\frac{a\;q^{2}\;sgn\left(\frac{l_{51}\;\lambda }{q}\right)}{l_{51}\;\lambda } & 0 & 0 & 0 & 0 & 0 \end{array}\right).$$

As there is a column of zeros, there will be at least one zero eigenvalue, meaning that this equilibrium is also non-hyperbolic. The eigenvector corresponding to the zero eigenvalue will be $\left(0,N_{2}^{*},0,0,0,0,0,0,0,0\right)$.

Upon evaluating the Jacobian at the steady state when $l_{12}+l_{23}=l_{34}+l_{45}+l_{51}$, $J_{E_{3}}$ the expression is given by

(A 62)
$$J_{E_{3}}=\left(\begin{array}{cccccccccc} -\frac{a\;q}{2\;l_{12}\;\lambda }-\frac{a\;q}{2\;l_{51}\;\lambda } & \frac{a\;q}{2\;l_{12}\;\lambda } & 0 & 0 & \frac{a\;q}{2\;l_{51}\;\lambda } & -\frac{\lambda }{q} & 0 & 0 & 0 & -\frac{\frac{l_{12}\;\lambda }{q}+\frac{l_{23}\;\lambda }{q}-\frac{l_{34}\;\lambda }{q}-\frac{l_{45}\;\lambda }{q}}{l_{51}}\\ \frac{a\;q}{2\;l_{12}\;\lambda } & -\frac{a\;l_{23}\;q}{2\;\lambda }-\frac{a\;q}{2\;l_{12}\;\lambda } & \frac{a\;l_{23}\;q}{2\;\lambda } & 0 & 0 & \frac{\lambda }{q} & -\frac{{l_{23}}^{2}\;\lambda }{q} & 0 & 0 & 0\\ 0 & \frac{a\;q}{2\;l_{23}\;\lambda } & -b-\frac{a\;q}{2\;l_{23}\;\lambda }-\frac{a\;q}{2\;l_{34}\;\lambda } & \frac{a\;q}{2\;l_{34}\;\lambda } & 0 & 0 & \frac{\lambda }{q} & \frac{\lambda }{q} & 0 & 0\\ 0 & 0 & \frac{a\;q}{2\;l_{34}\;\lambda } & -\frac{a\;l_{45}\;q}{2\;\lambda }-\frac{a\;q}{2\;l_{34}\;\lambda } & \frac{a\;l_{45}\;q}{2\;\lambda } & 0 & 0 & -\frac{\lambda }{q} & \frac{{l_{45}}^{2}\;\lambda }{q} & 0\\ \frac{a\;q}{2\;l_{51}\;\lambda } & 0 & 0 & \frac{a\;l_{45}\;q}{2\;\lambda } & -\frac{a\;l_{45}\;q}{2\;\lambda }-\frac{a\;q}{2\;l_{51}\;\lambda } & 0 & 0 & 0 & -\frac{{l_{45}}^{2}\;\lambda }{q} & \frac{\frac{l_{12}\;\lambda }{q}+\frac{l_{23}\;\lambda }{q}-\frac{l_{34}\;\lambda }{q}-\frac{l_{45}\;\lambda }{q}}{l_{51}}\\ \frac{a\;q^{2}\;sgn\left(\frac{l_{12}\;\lambda }{q}\right)}{2\;l_{12}\;\lambda } & -\frac{a\;q^{2}\;sgn\left(\frac{l_{12}\;\lambda }{q}\right)}{2\;l_{12}\;\lambda } & 0 & 0 & 0 & 0 & 0 & 0 & 0 & 0\\ 0 & \frac{a\;q^{2}\;sgn\left(\frac{l_{23}\;\lambda }{q}\right)}{2\;l_{23}\;\lambda } & -\frac{a\;q^{2}\;sgn\left(\frac{l_{23}\;\lambda }{q}\right)}{2\;l_{23}\;\lambda } & 0 & 0 & 0 & 0 & 0 & 0 & 0\\ 0 & 0 & -\frac{a\;q^{2}\;sgn\left(\frac{l_{34}\;\lambda }{q}\right)}{2\;l_{34}\;\lambda } & \frac{a\;q^{2}\;sgn\left(\frac{l_{34}\;\lambda }{q}\right)}{2\;l_{34}\;\lambda } & 0 & 0 & 0 & 0 & 0 & 0\\ 0 & 0 & 0 & -\frac{a\;q^{2}\;sgn\left(\frac{l_{45}\;\lambda }{q}\right)}{2\;l_{45}\;\lambda } & \frac{a\;q^{2}\;sgn\left(\frac{l_{45}\;\lambda }{q}\right)}{2\;l_{45}\;\lambda } & 0 & 0 & 0 & 0 & 0\\ \frac{a\;q^{2}\;sgn\left(\frac{l_{12}\;\lambda }{q}+\frac{l_{23}\;\lambda }{q}-\frac{l_{34}\;\lambda }{q}-\frac{l_{45}\;\lambda }{q}\right)}{2\;l_{51}\;\lambda } & 0 & 0 & 0 & -\frac{a\;q^{2}\;sgn\left(\frac{l_{12}\;\lambda }{q}+\frac{l_{23}\;\lambda }{q}-\frac{l_{34}\;\lambda }{q}-\frac{l_{45}\;\lambda }{q}\right)}{2\;l_{51}\;\lambda } & 0 & 0 & 0 & 0 & \frac{q\;\left|\frac{l_{12}\;\lambda }{q}+\frac{l_{23}\;\lambda }{q}-\frac{l_{34}\;\lambda }{q}-\frac{l_{45}\;\lambda }{q}\right|}{l_{51}}-\lambda \end{array}\right).$$

This expression can be further simplified to:

(A 63)
$$J_{E_{3}}=\left(\begin{array}{cccccccccc} -\frac{a\;q}{2\;l_{12}\;\lambda }-\frac{a\;q}{2\;l_{51}\;\lambda } & \frac{a\;q}{2\;l_{12}\;\lambda } & 0 & 0 & \frac{a\;q}{2\;l_{51}\;\lambda } & -\frac{\lambda }{q} & 0 & 0 & 0 & -\frac{\lambda }{q}\\ \frac{a\;q}{2\;l_{12}\;\lambda } & -\frac{a\;l_{23}\;q}{2\;\lambda }-\frac{a\;q}{2\;l_{12}\;\lambda } & \frac{a\;l_{23}\;q}{2\;\lambda } & 0 & 0 & \frac{\lambda }{q} & -\frac{{l_{23}}^{2}\;\lambda }{q} & 0 & 0 & 0\\ 0 & \frac{a\;q}{2\;l_{23}\;\lambda } & -b-\frac{a\;q}{2\;l_{23}\;\lambda }-\frac{a\;q}{2\;l_{34}\;\lambda } & \frac{a\;q}{2\;l_{34}\;\lambda } & 0 & 0 & \frac{\lambda }{q} & \frac{\lambda }{q} & 0 & 0\\ 0 & 0 & \frac{a\;q}{2\;l_{34}\;\lambda } & -\frac{a\;l_{45}\;q}{2\;\lambda }-\frac{a\;q}{2\;l_{34}\;\lambda } & \frac{a\;l_{45}\;q}{2\;\lambda } & 0 & 0 & -\frac{\lambda }{q} & \frac{{l_{45}}^{2}\;\lambda }{q} & 0\\ \frac{a\;q}{2\;l_{51}\;\lambda } & 0 & 0 & \frac{a\;l_{45}\;q}{2\;\lambda } & -\frac{a\;l_{45}\;q}{2\;\lambda }-\frac{a\;q}{2\;l_{51}\;\lambda } & 0 & 0 & 0 & -\frac{{l_{45}}^{2}\;\lambda }{q} & \frac{\lambda }{q}\\ \frac{a\;q^{2}\;}{2\;l_{12}\;\lambda } & -\frac{a\;q^{2}\;}{2\;l_{12}\;\lambda } & 0 & 0 & 0 & 0 & 0 & 0 & 0 & 0\\ 0 & \frac{a\;q^{2}\;}{2\;l_{23}\;\lambda } & -\frac{a\;q^{2}\;}{2\;l_{23}\;\lambda } & 0 & 0 & 0 & 0 & 0 & 0 & 0\\ 0 & 0 & -\frac{a\;q^{2}\;}{2\;l_{34}\;\lambda } & \frac{a\;q^{2}\;}{2\;l_{34}\;\lambda } & 0 & 0 & 0 & 0 & 0 & 0\\ 0 & 0 & 0 & -\frac{a\;q^{2}\;}{2\;l_{45}\;\lambda } & \frac{a\;q^{2}\;}{2\;l_{45}\;\lambda } & 0 & 0 & 0 & 0 & 0\\ \frac{a\;q^{2}\;}{2\;l_{51}\;\lambda } & 0 & 0 & 0 & -\frac{a\;q^{2}\;}{2\;l_{51}\;\lambda } & 0 & 0 & 0 & 0 & 0 \end{array}\right).$$

The determinant of $J_{E_{3}}$ is given by

(A 64)
$$\det\left(J_{E_{3}}\right)=-\frac{a^{4}\;q^{3}\left(b\;l_{23}\;l_{45}\;q\;\left|l_{12}\;\lambda +l_{23}\;\lambda -l_{34}\;\lambda -l_{45}\;\lambda \right|-b\;l_{23}\;l_{45}\;l_{51}\;\lambda \;q\right)}{16\;l_{12}\;l_{34}\;l_{51}}.$$

At the equilibrium point $E_{3}$, we know that both paths possess equal lengths, implying that

(A 65)
$$\begin{array}{lcr} & \left|l_{12},\lambda +l_{23},\lambda -l_{34},\lambda -l_{45},\lambda \right|=l_{51}. & \end{array}$$

Substituting equality equation (A 65) into (A 64), we find that the determinant becomes zero, indicating that $J_{E_{3}}$ also corresponds to a non-hyperbolic equilibrium.

## B. Further results

### B.1. Non-oscillatory sources and sinks

In this section, we include more results from the simulations on cycle graphs of larger sizes and more general graph structure; see figures 11–13.

We observe that the slime mould model can still find the shortest path in both cases.

### B.2. Oscillating nodes

See figures 14 and 15
